# Women’s reproductive autonomy during the COVID-19 pandemic: a cross-sectional study^*^

**DOI:** 10.1590/1518-8345.6910.4437

**Published:** 2025-01-31

**Authors:** Carolyne Souza de Moura Barbosa, Ana Cecília Silvestre da Silva, Géssyca Cavalcante de Melo, Alba Maria Bomfim de França

**Affiliations:** 1 Universidade Estadual de Ciências da Saúde de Alagoas, Maceió, AL, Brazil.; 2 Scholarship holder at the Fundação de Amparo à Pesquisa do Estado de Alagoas (FAPEAL), Brazil.; 3 Universidade Estadual de Ciências da Saúde de Alagoas, Núcleo de Saúde Materno-Infantil e do Adolescente, Maceió, AL, Brazil.; 4 Maternidade Escola Santa Mônica, Unidade de Terapia Intensiva Neonatal, Maceió, AL, Brazil.; 5 Universidade Estadual de Ciências da Saúde de Alagoas, Núcleo de Ensino de Propedêutica e Terapêutica, Maceió, AL, Brazil.; 6 Secretaria Municipal de Saúde de Maceió, Diretoria de Vigilância em Saúde, Maceió, AL, Brazil.; 7 Centro Universitário de Maceió, Maceió, AL, Brazil.

**Keywords:** Personal Autonomy, Reproductive Behavior, Women’s Health, Contraceptives Agents, Family Planning, COVID-19

## Abstract

**Objective:**

to analyze the reproductive autonomy of women during the COVID-19 pandemic, considering sociodemographic, clinical, and reproductive factors.

**Method:**

a quantitative study with a cross-sectional design, conducted with 314 women aged 18 to 49 years old. Data were collected through an online questionnaire containing sociodemographic, clinical, and reproductive data, as well as the Reproductive Autonomy Scale. The Mann-Whitney and Student’s t tests were used to compare variables.

**Results:**

significant differences were found between the average scores of “decision-making” and marital status (*p* = <0.001); and “absence of coercion” and “communication” with age group (*p* = 0.03 e <0.001), residence (*p* = <0.001 and <0.01), schooling level (*p* = 0.02 e 0.02), pregnancy (*p* = <0.001 e 0.04) and contraception (*p* = 0.02 e <0.001).

**Conclusion:**

not having a sexual partner positively influenced autonomy in reproductive decision-making during the COVID-19 pandemic. Women of younger age, living in the capital, with higher education levels, who had never been pregnant, and who used contraceptives during the pandemic showed greater autonomy in the absence of coercion and communication. It was possible to identify the groups that require greater attention and interventions to support their sexual health and reproductive choices.

## Highlights

**(1)** A high reproductive autonomy score was observed even during the pandemic.

**(2)** Not having a stable partner positively influenced reproductive decision-making.

**(3)** Women living in the capital, of younger age, and with lower education levels experienced less coercion.

**(4)** Being nulligravida and using contraception were associated with less coercion and better communication.

**(5)** The use of an online tool during the pandemic expanded the program’s reach to women.

## Introduction

Women’s reproductive rights were achieved after social and feminist mobilizations that began in the 1960s, transforming sexuality and benefiting their social status. However, despite these victories, the limits imposed by gender inequality persist in women’s daily lives and continue to interfere with their reproductive autonomy^(1)^.

Ensuring reproductive autonomy is a complex task that involves multiple factors, considering women’s life context and individual needs, as well as socioeconomic and demographic factors such as age, residence, years of schooling, religion, marital status, race, among other health determinants and conditions^(2-3)^.

Gender differences can also influence whether or not to choose contraception, considering women’s greater knowledge about available contraceptive methods and the strong male influence in reproductive planning^(4)^. The life context must be taken into account when choosing contraception, including partner participation in this process, as well as the emancipation and reproductive autonomy of women^(2)^.

The importance of access to modern contraception for women and their sexual partners is emphasized, as well as the freedom to choose it, provided it is acceptable and safe based on their specific clinical conditions, as assessed during consultation with a health care professional. For those who do not wish to become pregnant, starting or continuing the chosen contraceptive method ensures their right to sexual and reproductive health^(5)^. Reproductive autonomy grants women the full right to take charge of their contraceptive choices, whether to have or not have children, and to use or not use contraception^(6)^.

The SARS-CoV-2 (COVID-19) pandemic, particularly during the period from 2020 to 2021, imposed numerous restrictions on the population due to the need for social distancing to prevent and reduce the spread of infection. These restrictions also impacted the availability of health care professionals, in addition to causing illness and occupational stress, which affected the quality and responsiveness of health services^(7)^.

In the context of reproductive planning, physical distancing, remote work, and restrictions on non-essential activities hindered access to sexual and reproductive health services. This may have affected women’s rights and autonomy, making them more prone to unplanned pregnancies^(8-9)^.

Access to contraception positively impacts the lives, health, and well-being of women and their families, in addition to reducing unplanned pregnancies^(10)^. Reproductive autonomy among Brazilian women ranges from medium^(11)^ to high levels^(12)^, yet challenges persist regarding socioeconomic factors and the partner’s role in the reproductive decision-making process. Thus, gender roles and power dynamics in decision-making are gaps that warrant further discussion^(6)^, as are socioeconomic and cultural factors, which are highlighted with equal importance and influence in a study conducted in a developing country, which focused on adolescents and women, emphasizing the presence of stigma as an element that requires further exploration^(13)^, which remains a gap in studies involving Brazilian women.

The violation of reproductive rights and social impositions undermine autonomy and can hinder Brazilian women in achieving their reproductive goals^(11)^. Thus, the choice of this topic was motivated by the opportunity to reflect on women’s reproductive autonomy during the COVID-19 pandemic, taking into account the changes brought about by this period and the influence of social determinants of health.

This study aims to contribute to a better understanding of reproductive autonomy and whether it was preserved during the COVID-19 pandemic. Therefore, its objective was to analyze the reproductive autonomy of women during the COVID-19 pandemic, considering sociodemographic, clinical, and reproductive factors.

## Method

### Study design

This is a quantitative study with a cross-sectional design.

### Setting

The study was conducted across the state of Alagoas, Brazil. Located in the northeastern region and composed of 102 municipalities, Alagoas has an estimated population of 3,365,351 inhabitants and is divided into two health macro-regions. The first macro-region covers the first to the sixth health regions, totaling 57 municipalities (2,276,293 inhabitants), including the capital, Maceió. Meanwhile, the second health macro-region covers the seventh to the tenth regions, totaling 45 municipalities (1,089,058 inhabitants)^(14)^.

### Period

Data collection was carried out from June 24 to November 30, 2021.

### Population

The study population consisted of women residing in the state of Alagoas, aged between 18 and 49 years old.

### Selection criteria

Inclusion criteria were women aged 18 to 49 years old and residents of the state of Alagoas. Exclusion criteria: women whose data collection instruments were incomplete, duplicated, or lacked email or WhatsApp^®^ contact information for sending the Informed Consent Form (ICF).

### Definition of the sample

To obtain the study sample, a search was conducted in the Health Informatics Department of the Unified Health System (DATASUS, its acronym in Portuguese) regarding the female population residing in the state of Alagoas, of childbearing age, categorized by age group from 18 to 49 years old, and by health macro-region, based on data from 2010, the year of the last census conducted by the Brazilian Institute of Geography and Statistics (*Instituto Brasileiro de Geografia e Estatística* - IBGE).

The research covered a total of 777,970 women, with 536,820 women in the first health macro-region and 241,150 women in the second health macro-region. From this, the study sample was estimated using sample calculation, with a 5% margin of error and a 95% confidence level, resulting in a total of 384 participants.

After applying the inclusion and exclusion criteria, a total of 314 women were included, whose instruments were complete, using as a reference a study conducted in northeastern Brazil^(12)^. To minimize sampling bias, a targeted outreach strategy was used in municipalities with fewer respondents to the online information collection instruments It is worth noting that initially, 404 women participated in the study. After excluding incomplete instruments (n=62), those answered by the same participant (n=12), those with incompatible responses to the age question (n=14), and those without an email or WhatsApp^®^ contact for sending the ICF (n=2), the final sample consisted of 314 women.

### Study variables

Sociodemographic, clinical, and reproductive data were collected using 46 variables, which were used to characterize the participants. Sociodemographic data: city, age, race (self-declared), current sexual partnership, religion, monthly income, participant’s schooling level and head of household’s schooling level, whether they have health insurance, and if they receive government assistance. To aid in data analysis, the alternatives for the variable “current partnership” were synthesized into two groups: without a partner (single, widowed, or divorced) or with a sexual partner (married or in a stable union).

Among the sociodemographic variables, 15 were added based on the Brazilian Economic Classification Criteria (*Critério Brasil*) of the Brazilian Association of Research Companies^(15)^, which assesses Brazil’s economic classification and consists of variables with response options: zero, one, two, three, or four or more. These include: the number of bathrooms, monthly-employed domestic workers who work five or more days a week, and personal or family-use cars in the participant’s household; the number of personal computers, dishwashers, washing machines, dryers, refrigerators, freezers, Digital Versatile Disc (DVD) players, microwaves, and motorcycles in the participant’s household; whether the household has running water and whether the street where the participant lives is paved.

Each response (zero, one, two, three, or four or more), depending on the variable, corresponds to a number of points ranging from 0 to 14. A total score is obtained by summing the points for each variable, resulting in a classification according to the Brazilian Economic Classification Criteria based on average income: Class A (BRL 21,826.74) = 45 to 100 points; Class B1 (BRL 10,361.48) = 38 to 44 points; Class B2 (BRL 5,755.23) = 29 to 37 points; Class C1 (BRL 3,276.76) = 23 to 28 points; Class C2 (BRL 1,965.87) = 17 to 22 points; and Class DE (BRL 900.60) = 0 to 16 points. The highest average income corresponds to Class “A” and the lowest to Class “DE” decreasing progressively^(15)^. The other sociodemographic variables were developed by the authors, as were the clinical and reproductive variables.

Clinical conditions data: presence of dyslipidemia, hypertension, diabetes, obesity, sedentary lifestyle, smoking, or others. Reproductive data: number of pregnancies, including miscarriages; whether the participant has children, how many, the spacing between them, and if they still plan to have more; pregnancy planning; contraceptive methods known, previously used, used before the COVID-19 pandemic, currently in use, and preferred methods; how long the current contraceptive method has been in use; whether the participant sought reproductive planning services or professional counseling during the pandemic (reproductive counseling); from whom they received contraceptive advice; how they accessed contraceptives, whether they pay for them, and whether the cost affects their income; if they believe their contraceptive choices were impacted by the pandemic; if they experienced difficulties accessing contraceptives before or during the pandemic; whether they stopped using contraceptives during the pandemic, and the reason for discontinuing them.

Reproductive autonomy was assessed using the Reproductive Autonomy Scale, which was translated, validated, and adapted for Brazilian women^(16)^. It includes 14 variables organized into three subscales, which are detailed in the section on the instruments used for data collection.

### Instruments used for data collection

The data were collected through a questionnaire for assessing sociodemographic, clinical, and reproductive data, and the Reproductive Autonomy Scale — Brazilian version^(16)^. The questionnaire consists of 46 questions. The Brazilian version of the Reproductive Autonomy Scale^(16)^ consists of 14 items, organized into three subscales: decision-making, absence of coercion, and communication.

The decision-making subscale includes four questions about who has the final say in reproductive situations: (1) Who decides whether you use a method to prevent pregnancy?; (2) Who decides which method you will use to prevent pregnancy?; (3) Who decides when you will have a baby?; (4) If you had an unplanned pregnancy, who would decide what to do — whether to raise the child, seek adoptive parents, or have an abortion? For this subscale, a score is obtained based on the responses: My sexual partner = 1 point; Both my partner and I equally = 2 points; I = 3 points Participants are instructed to consider “sexual partner” as their main or most recent partner, or even a family member who might influence the decision, such as a parent or in-law^(16-17)^.

The absence of coercion subscale consists of five questions regarding coercive situations experienced by women: (1) Has your partner ever stopped you from using a method to prevent pregnancy when you wanted to?; (2) Has your partner ever hindered or made it difficult for you to use a method to prevent pregnancy when you wanted to?; (3) Has your partner ever made you use a method to prevent pregnancy when you did not want to?; (4) Would your partner stop you from using a method to prevent pregnancy if you wanted to?; (5) Has your partner ever pressured you to become pregnant^(16)^?

The communication subscale consists of five statements related to communication about the sexual relationship and reproductive decisions: (1) Would your partner support you if you wanted to use a method to prevent pregnancy?; (2) Is it easy to talk about sex with your partner?; (3) If you did not want to have sexual relations, could you tell your partner?; (4) If you were unsure about being pregnant or not, could you talk to your partner about it?; (5) If you really did not want to become pregnant, could you convince your partner not to have a child?^(16)^.

For the second and third subscales, responses follow a Likert scale (strongly disagree, disagree, agree, or strongly agree), with scores ranging from 1 to 4 points. For the second subscale, it is necessary to reverse the item scores to calculate the absence of coercion score, as all items are theoretically opposed to reproductive autonomy^(16)^.

After summing the three subscale scores for reproductive autonomy, an average score is computed for the scale. Higher average scores indicate higher levels of reproductive autonomy^(16-17)^. The total reproductive autonomy scale score ranges from 1.00 to 4.00, with a mean of 1.00 to 2.00 indicating low autonomy, 2.01 to 3.00 indicating medium autonomy, and 3.01 to 4.00 indicating high autonomy^(11-12)^.

### Data collection

Data collection was conducted remotely due to the COVID-19 pandemic, enabling access to women in all municipalities through the Google Forms tool. Invitations were sent via social media platforms Instagram^®^, Facebook^®^, email, and WhatsApp^®^. Promotional cards were created, featuring key information to capture the invitees’ attention and encourage them to continue reading the attached text. This text included an introduction to the researchers, the study’s objectives, the target population, and a link to the data collection instruments.

Instagram^®^ allows the sharing of photos, videos, and messages. Besides using the researchers’ personal accounts, a specific profile was created for promoting the study and inviting participants. Invitations were shared through stories, posts in the feed, and Direct messages. Additionally, to reach as many women as possible, Direct messaging was used to contact active profiles with a significant number of followers, targeting the intended audience and directing content to residents of various municipalities in Alagoas. Moreover, contact was made via WhatsApp^®^ with profile administrators who had made their phone numbers available in their Instagram^®^ bio.

On Facebook^®^, the cards and attached texts were shared through the researchers’ personal accounts. An institutional email was used to facilitate access to a wide range of student and faculty email addresses, whether known or unknown, from the university where the researchers studied and worked. WhatsApp^®^ is an application used for the same purpose as Instagram^®^, with promotions shared via group chats and individual messages.

It is important to note that all social media platforms were used daily for promotion throughout the data collection period, with an additional message requesting recipients to share the content. No gender distinction was made in the invitations; the message included information on the importance of sharing it with women who met the criteria if the recipient was not part of the target audience. In the final month of data collection, invitations were directed to municipalities with fewer respondents to the data collection instruments.

Participants filled out the online questionnaire, which collected sociodemographic, clinical, and reproductive data, and completed the Brazilian version of the Reproductive Autonomy Scale^(16)^. This process was carried out at times convenient for the participants.

Upon accessing the Google Forms link through the open invitation, participants read and acknowledged the study’s nature. They agreed to participate in the study by clicking the “I AGREE” button on the ICF which was individually sent along with a copy of the responses to the email or WhatsApp^®^ number provided at the beginning of the data collection instrument, for archiving and/or printing purposes.

Immediately after, the participants completed the data collection instruments for the research. The average response time was 15 to 20 minutes. In case of doubts, participants could contact the researchers via email. A non-probabilistic recruitment technique was used, where participants were included once they agreed to participate in the study and then underwent the exclusion criteria.

### Data treatment and analysis

The data were imported into Microsoft Excel for Windows for organization and to present the results of the means, standard deviations, and minimum and maximum values, using descriptive statistics. For the comparison of the means of the domains of the Brazilian Version of the Reproductive Autonomy Scale between groups, the Mann-Whitney test was used for non-parametric data, and the Student’s t-test was used for parametric data. Data normality was assessed using the Shapiro-Wilk test, and homoscedasticity was evaluated with Levene’s test. A significance level of *p* < 0.05 was considered. The analyses were performed using Jeffreys’s Amazing Statistics Program (JASP) version 0.16.1.

### Ethical aspects

The study adhered to all ethical guidelines in accordance with Resolution No. 466 of 2012 and Resolution No. 510 of 2016 of the National Research Ethics Council (CONEP). The research was approved under opinion 4.794.176 on June 21, 2021. To address any questions regarding the topic, a link was provided at the end of the data collection process, redirecting participants to a guide on Women’s Sexual and Reproductive Rights During the COVID-19 Pandemic. This guide was prepared by the Public Defender’s Office of the State of São Paulo and the Specialized Center for the Promotion and Defense of Women’s Rights (NUDEM) and contains guidelines on contraception, legal abortion, pregnancy, childbirth, and postpartum care^(18)^.

## Results

The sociodemographic, clinical, and reproductive characteristics of the study participants (n=314) were evaluated. The analysis of sociodemographic characteristics showed that the average age of the women was 27.2 years. Additionally, 87.26% (n=186) lived in the capital of Alagoas; 87.26% (n=274) self-identified as non-Black, with 37.58% (n=118) identifying as white, 46.18% (n=145) as brown (mixed race), 3.18% (n=10) as yellow (Asian), and 0.32% (n=1) as indigenous; 12.74% (n=40) identified as Black; and 85.03% (n=267) of the women had a religious affiliation.

Most of the study sample consisted of women without a sexual partner, that is, single, widowed, or divorced, totaling 64.97% (n=204). It was identified that 98.41% (n=309) of the participants had more than eight years of schooling, and 54.78% (n=172) of the sample had a monthly income higher than one minimum wage. Specifically, 25.16% (n=79) earned up to two minimum wages, 18.79% (n=59) earned between three and five minimum wages, and 10.83% (n=34) earned more than five minimum wages, leaving 45.22% (n=142) of the women with an income of up to one minimum wage. According to the Brazilian Economic Classification Criterion^(12)^, 32.80% (n=103) of the women belonged to class “B2” (average income BRL 5,755.23), followed by class “C1” (average income BRL 3,276.76) with 19.11% (n=60). A minority belonged to class “A” (average income BRL 21.826,74) with 6.69% (n=21) and class “DE” (average income BRL 900.60) with 7.32% (n=23).

Among the clinical conditions reported by 21.19% (n=68) of the women, 0.94% (n=03) had dyslipidemia, 3.43% (n=11) had arterial hypertension, 0.31% (n=01) had diabetes, 4.98% (n=16) had obesity, 7.17% (n=23) reported a sedentary lifestyle, 0.94% (n=03) smoked, and 3.43% (n=11) had other conditions. As a result, 78.82% (n=253) of the women reported no clinical alteration.

Regarding reproductive characteristics, 64.65% (n=203) of the participants had never been pregnant (including miscarriage), and 20.70% (n=65) of those who had been pregnant reported an unplanned pregnancy. Furthermore, 81.53% (n=256) used contraceptive methods during the COVID-19 pandemic.

The most commonly used contraceptive methods during the pandemic were hormonal, with 60.51% (n=190) using it, followed by 41.72% (n=131) using barrier methods and 28.98% (n=91) using behavioral methods. A total of 45.22% (n=142) of the participants had been using their contraceptive method for more than two years. The majority of the participants did not discontinue the use of contraceptives during the COVID-19 pandemic, totaling 74.52% (n=234) of the sample.

Additionally, 61.47% (n=193) of the women accessed contraceptives by purchasing them themselves, and 87.26% (n=274) did not experience difficulty in obtaining contraceptives during the pandemic However, 67.20% (n=211) did not attend any reproductive planning services or receive professional counseling during the COVID-19 pandemic.

Table 1 presents the means, standard deviations, and minimum and maximum scores for the domains of reproductive autonomy. The highest levels of autonomy were observed in the “Absence of Coercion” and “Communication” constructs, while the lowest levels of autonomy were seen in “Decision-Making.”

**Table 1:** - Reproductive autonomy scores of the study participants, categorized by each domain of the Reproductive Autonomy Scale (N^*^ = 314). Maceió, AL, Brazil, 2021

**Factor (subscale)**	**Mean**	**Standard Deviation**	**Minimum-Maximum**
Decision-making	2.65	0.51	1.00 - 3.00
Absence of coercion	3.63	0.66	1.00 - 4.00
Communication	3.41	0.75	1.00 - 4.00
Total	3.27	0.77	1.00 - 4.00

^*^N = Total number of participants

The mean scores of reproductive autonomy and the sociodemographic and reproductive characteristics of the study participants were analyzed, as presented in Table 2. A statistically significant difference was found between the mean scores of “Absence of Coercion” and “Communication” concerning age. According to the analyses, women aged 18 to 35 years old showed greater autonomy in both scores compared to women older than 35 years.

**Table 2:** - Comparison of mean reproductive autonomy scores and sociodemographic and reproductive characteristics of study participants (N^*^ = 314). Maceió, AL, Brazil, 2021

**Variables**	**Decision-making**	**Absence of coercion**	**Communication**
**Age group (years old)**			
18-35	2.66±0.35	3.65±0.55	3.45±0.50
> 35	2.59±0.41	3.50±0.50	3.19±0.61
*p*-value	0.37	*0.03*	*<0.00* ^†^
**Home**			
Capital	2.68±0.33	3.70±0.51	3.48±0.49
Inland	2.61±0.38	3.52±0.59	3.31±0.55
*p*-value	0.12	*<0.001* ^†^	*<0.01* ^†^
**Self-declared skin color**			
Black	2.73±0.34	3.56±0.57	3.40±0.43
Non-Black	2.64±0.36	3.64±0.55	3.41±0.53
*p-*value	0.11	0.30	0.60
**Religion**			
Religious affiliation	2.64±0.36	3.61±0.55	3.39±0.53
No religion	2.72±0.35	3.69±0.54	3.51±0.44
*p-*value	0.07	0.23	0.21
**Current sexual partnership**			
With sexual partnership	2.54±0.38	3.64±0.52	3.41±0.58
No sexual partnership	2.71±0.33	3.62±0.57	3.41±0.49
*p*-value	*<0.001*	0.96	0.56
**Income**			
Up to 1 minimum wage^‡^	2.66±0.37	3.58±0.59	3.35±0.50
> 1 minimum wage^‡^	2.64±0.34	3.67±0.51	3.46±0.54
*p-*value	0.36	0.31	0.08
**Schooling**			
≥ 8 years of study	2.75±0.43	2.92±0.81	2.72±0.77
> 8 years of study	2.65±0.36	3.64±0.54	3.42±0.51
*p*-value	0.38	*0.02*	*0.02*
**Pregnancy**			
No	2.65±0.35	3.70±0.49	3.46±0.50
Yes	2.66±0.38	3.49±0.63	3.33±0.55
*p*-value	0.56	*<0.001* ^†^	*0.04* ^†^
**Contraception**			
No	2.68±0.39	3.51±0.56	3.14±0.63
Yes	2.65±0.35	3.65±0.55	3.47±0.47
*p-*value	0.31	*0.02* ^†^	*<0.001*
**Reproductive counseling**			
No	2.66±0.36	3.62±0.56	3.37±0.55
Yes	2.62±0.35	3.63±0.53	3.50±0.45
*p-*value	0.22	0.93	0.09

The values are expressed as mean ± standard deviation; ^*^N = Total number of participants; ^†^Student’s t-test; ^‡^The minimum wage at the time of data collection was BRL 1,100.00, Brazil, 2021

A statistically significant difference between the mean scores of “Absence of coercion” and “Communication” in relation to residence was also observed. The analyses indicated that participants living in the capital of Alagoas exhibited higher reproductive autonomy than those living in rural areas.

Regarding sociodemographic characteristics, a statistically significant difference was found between the mean score of “Decision-making” and the current sexual partnership. The analyses showed that women without a partner (single, widowed, and divorced) demonstrated higher autonomy in the “Decision-making” construct compared to participants with a sexual partner (married and in stable unions).

A statistically significant difference between the mean scores of “Absence of coercion” and “Communication” with schooling level was also found. According to the analyses, participants with more than eight years of education demonstrated greater autonomy in the “Absence of Coercion” and “Communication” constructs compared to those with up to eight years of education.

In terms of reproductive characteristics, a statistically significant difference was found between the mean scores of “Absence of coercion” and “Communication” with pregnancy and contraception. According to the analyses, women who had never been pregnant (including those who had abortions) and who used contraceptive methods during the COVID-19 pandemic demonstrated greater reproductive autonomy compared to women who had been pregnant (including those who had abortions) and did not use contraceptives.

## Discussion

During the data collection period, the COVID-19 pandemic was still ongoing in Brazil. In Alagoas, the number of confirmed cases and deaths was rising, with an incidence rate of 6.3% in June and 7.2% in November 2021^(19-20)^. However, there was some easing of restrictions and vaccinations available for individuals over 18 years old starting in August 2021^(21)^, along with the gradual reopening of establishments with occupancy limits and proof of vaccination required^(22)^. Beyond the impacts of the COVID-19 pandemic, it is important to consider that the challenges and difficulties women face in exercising their full reproductive autonomy depend on several associated factors, such as their sociodemographic and reproductive context^(6)^.

The women in this study demonstrated a high average score for overall reproductive autonomy. The highest autonomy was observed in the constructs “Absence of Coercion” and “Communication,” while the lowest was seen in “Decision-Making.” This finding differs from other Brazilian studies that reported greater autonomy in “Decision-Making”^(11)^ and lower autonomy in “Communication”^(12)^. Conversely, it aligns with a study of American women, which also indicated lower autonomy in “Decision-Making”^(17)^.

It is well established that sociodemographic factors contribute to women’s reproductive autonomy^(6)^. In this study, regarding the “Decision-Making” subscale and the variable of current sexual partnership, it was observed that women without a sexual partner (single, widowed, and divorced) showed higher reproductive autonomy. This finding is consistent with a study of Afro-Brazilian *quilombola* women^(11)^ but contrasts with research conducted with Brazilian rural workers^(12)^. Furthermore, a study on Nigerian women revealed that most participants had health decisions made by their partners, with factors such as wealth index and education level associated with this dynamic^(23)^. Women’s reproductive intentions are shaped by their relationships with sexual partners, including partner interference with contraception and experiences of reproductive coercion, revealing submission to partner decisions, which are reinforced by patriarchal structures and surrounding cultural norms^(17)^. The male pressure and coercion reinforce gender hierarchy and are present from the onset of sexual activity, during which women are still building their sexual autonomy^(24)^. The majority of female submission in decision-making is related to the number of children a couple has and financial dependency, even though contraceptive responsibility is placed primarily on women^(4)^.

The age variable demonstrated that women between the ages of 18 and 35 years old exhibit higher reproductive autonomy in the “Absence of Coercion” and “Communication” constructs compared to women over 35, similar to findings from studies that validated the reproductive autonomy scale and indicated this data among younger women^(17)^. Considering that the sample includes women with an average age of 27.2 years old, younger couples may have more effective communication regarding reproductive decisions, leading to greater reproductive autonomy.

Additionally, the increasingly early onset of sexual activity, reliance on partner trust as a prerequisite for condom use, and the risks faced by more vulnerable populations regarding unplanned pregnancies are factors that may have long-term implications for the sexual and reproductive health of younger populations^(25)^. Conversely, sexually active women who do not use contraceptive methods often cite not wanting or not caring about becoming pregnant as the main reason, while younger women are more likely to practice dual protection^(3)^.

Regarding the residence variable, participants living in the capital of Alagoas demonstrated greater reproductive autonomy in the “Absence of Coercion” and “Communication” constructs compared to women living in the state’s rural areas. This finding is consistent with a study conducted in Brazil with rural women, which highlighted the challenges they face in achieving their reproductive goals due to social determinants^(12)^. These challenges may be tied to access to information for women with lower income, education, and housing conditions. The combination of unfavorable sociodemographic factors, communication difficulties with partners, and experienced coercion can lead to serious consequences for women’s health and well-being, ultimately hindering the development of reproductive autonomy. In line with this, a study with adolescents in Rio de Janeiro’s favelas revealed that the disregard and silencing of the sexual and reproductive choices among women in more vulnerable situations can result in unplanned pregnancies and unsafe abortions^(24)^.

In terms of education, women with more than eight years of schooling exhibited greater reproductive autonomy in the “Absence of Coercion” and “Communication” constructs compared to women with fewer than eight years of schooling. A study in Nigeria demonstrated that the higher a woman’s education level, the more capable she is of making her own health decisions without interference from her partner or joint decision-making^(23)^.

The knowledge of contraception and reproduction gained through higher education may have facilitated effective communication with partners in achieving reproductive goals, given that men are often afforded social authority in decision-making. It is important to note that low educational attainment and the resulting lack of knowledge among women reinforce gender hierarchies and male dominance in decision-making^(23)^.

Autonomy in the “Communication” subscale is consistently observed across studies^(12-13,17)^, highlighting that effective communication with sexual partners plays a significant role in reproductive decisions. The inability to assert one’s preferences, whether to refuse sexual activity or to request condom use, can undermine women’s sexual and reproductive rights^(24)^. Trust in a partner is an essential factor for regular condom use and influences risky sexual behaviors, driven by the duration and type of relationship^(26)^.

While no statistically significant differences were observed in the “race” variable, several studies evaluating its influence on reproductive autonomy suggest that Black women face greater limitations due to the effects of structural racism, which impacts their reproductive decisions^(12)^.

A study with Black women revealed lower autonomy in the “Absence of Coercion” and “Communication” subscales^(17)^ and another with *quilombola* women, 64.7% of whom self-identified as Black, highlighted the influence of sociodemographic and reproductive factors^(11)^. It is worth noting that only 12.74% of the participants in this study self-identified as Black, which may have contributed to the more favorable outcomes related to reproductive autonomy.

Regarding the variables of pregnancy and contraception, women who had never been pregnant (including miscarriages) and who used contraceptives during the COVID-19 pandemic exhibited greater reproductive autonomy in the “Communication” subscale compared to women who had been pregnant (including miscarriages) and who did not use contraceptives. Communication between couples is essential in all aspects of reproduction, including miscarriage. A study examining male perspectives on this issue highlights the differences in gender relations across different social classes and how women’s reproductive autonomy involves complex relationships with those around them, which can lead to varying levels of partner involvement in decisions about whether to carry a pregnancy to term or seek termination^(27)^.

As such, communication between couples is equally important for contraception. The ability to communicate with a sexual partner is a perceived benefit and a critical element in contraception and reducing risky behaviors^(26)^. It is important to consider male pressure in contraceptive choices and women’s autonomy^(4)^. The pregnancy and contraception variables also demonstrated greater reproductive autonomy in the “Absence of Coercion” construct among women who had never been pregnant (including miscarriages) and who used contraceptives during the COVID-19 pandemic.

The fact that most participants had been using hormonal contraceptives for over two years, purchased with their own money and without access difficulties, suggests autonomy and empowerment in reproductive decision-making. However, it is essential to recognize the privileged position of most participants, as education level, income, and housing conditions are contributing factors to contraceptive access. Despite the widespread access to hormonal methods, these women do not frequently use long-term contraceptive methods.

Additionally, it is important to highlight how patriarchal culture and societal judgments can affect women’s choices regarding motherhood, reinforced by societal expectations of what it means to be a woman and the belief that women are solely responsible for the household and their children^(28)^. In conclusion, maintaining the right to contraceptive access helps reduce unplanned pregnancies and significantly impacts women’s lives and reproductive autonomy^(10)^.

The discontinuity of health services and reduced access to contraceptive methods during the COVID-19 pandemic restrictions may have negative consequences for women’s health^(10)^. Limitations in access to information, services, and contraceptives do not contribute to enhancing women’s autonomy^(8)^.

It is noteworthy that shared decision-making between a woman and a healthcare professional can support informed choices and effective adherence to contraceptive methods, as long as it is done with care, respecting the woman’s autonomy and preferences^(29)^. In this context, although this study did not identify statistically significant differences regarding reproductive counseling, it is important to highlight the value of reproductive planning, especially considering that 67.20% of participants did not attend any service or professional counseling for that purpose during the COVID-19 pandemic. This lack of access could lead to risks such as unplanned pregnancies and lower adherence to more effective long-term methods.

The study presented some limitations regarding sample size, as 22% of the collected instruments were excluded due to incomplete responses (duplicates, incompatible answers, and lack of contact information for sending the ICF) despite the high level of education among participants. The use of an online questionnaire and scale, shared through social media and requiring internet access, may have posed challenges for women with lower levels of education and income. Additionally, the profiles of the study participants may not represent the general population.

The use of an online tool allowed for reaching women in remote areas and expanded the study’s reach across municipalities, considering the movement restrictions during the COVID-19 pandemic.

The study’s findings contribute to clarifying women’s autonomy during the pandemic and its relationship with their sexual partnerships, as well as comparing sociodemographic and reproductive factors, providing a basis for building and improving public policies aimed at addressing these factors.

The contributions of this study are particularly relevant to health care professionals who interact closely with the population, such as nurses, especially those involved in reproductive planning. It may also be of interest to professionals beyond the health care field, promoting changes in the culture of solely holding women accountable for contraception and advocating for strategic actions to include sexual partners in this process.

## Conclusion

This study assessed the reproductive autonomy of women during the COVID-19 pandemic. According to the data presented, not having a sexual partner (single, widowed, and divorced women) positively influenced reproductive decision-making autonomy during the pandemic. However, this same group showed lower overall reproductive autonomy in the “Decision-Making” subscale. Women of younger age, residing in the state capital, with higher education, who had never been pregnant, and who used contraceptives during the COVID-19 pandemic, exhibited greater autonomy in the areas of absence of coercion and communication.

Feeling comfortable discussing sexual and reproductive issues with their sexual partners can significantly influence women’s autonomy. The women in this study showed a high average score for total reproductive autonomy. The Brazilian version of the Reproductive Autonomy Scale contributed to understanding the reproductive intentions of the participating women, identifying groups needing more attention and suggesting interventions in sexual and reproductive health.
