# Supplementary material for: Women’s reproductive autonomy during the COVID-19 pandemic: a cross-sectional study
Source: Rev Lat Am Enfermagem. 2025 Jan 31;33:e4437. doi: 10.1590/1518-8345.6910.4437 (PMC11789717; doi:10.1590/1518-8345.6910.4437)
Supplement: Translation

# Translation: Autonomía reproductiva de las mujeres durante la pandemia de COVID-19: estudio transversal^*^

**DOI:** 10.1590/1518-8345.6910.4436

**Authors:** Carolyne Souza de Moura Barbosa, Ana Cecília Silvestre da Silva, Géssyca Cavalcante de Melo, Alba Maria Bomfim de França

**Affiliations:** 1 Universidade Estadual de Ciências da Saúde de Alagoas, Maceió, AL, Brasil.; 2 Becaria de la Fundação de Amparo à Pesquisa do Estado de Alagoas (FAPEAL), Brasil.; 3 Universidade Estadual de Ciências da Saúde de Alagoas, Núcleo de Saúde Materno-Infantil e do Adolescente, Maceió, AL, Brasil.; 4 Maternidade Escola Santa Mônica, Unidade de Terapia Intensiva Neonatal, Maceió, AL, Brasil.; 5 Universidade Estadual de Ciências da Saúde de Alagoas, Núcleo de Ensino de Propedêutica e Terapêutica, Maceió, AL, Brasil.; 6 Secretaria Municipal de Saúde de Maceió, Diretoria de Vigilância em Saúde, Maceió, AL, Brasil.; 7 Centro Universitário de Maceió, Maceió, AL, Brasil.

**Keywords:** Autonomía, Conducta Reproductiva, Salud de la Mujer, Anticonceptivos, Planificación Familiar, COVID-19

## Abstract

**Objetivo:**

analizar la autonomía reproductiva de las mujeres durante la pandemia de COVID-19, en función de factores sociodemográficos, clínicos y reproductivos.

**Método:**

estudio cuantitativo con diseño transversal, realizado con 314 mujeres de 18 a 49 años. La información se obtuvo mediante un cuestionario *online* que consta de datos sociodemográficos, clínicos y reproductivos y de la Escala de Autonomía Reproductiva. Para comparar variables se utilizaron las pruebas de Mann-Whitney y t de Student.

**Resultados:**

hubo una diferencia estadísticamente significativa entre los puntajes medios de “toma de decisiones” y estado civil (p = <0,001); y entre “ausencia de coerción” y “comunicación” y franja etaria (p = 0,03 y <0,001), residencia (p = <0,001 y <0,01), educación (p = 0,02 y 0,02), embarazo (p = <0,001 y 0,04) y anticoncepción (p = 0,02 y <0,001).

**Conclusión:**

no tener pareja sexual influyó positivamente en la autonomía en la toma de decisiones reproductivas durante la pandemia de COVID-19. Las mujeres de menor franja etaria, residentes en la capital, mayor nivel de escolaridad, que nunca habían estado embarazadas y que utilizaban anticonceptivos durante la pandemia mostraron mayor autonomía en ausencia de coerción y comunicación. Se identificaron grupos que requieren mayor atención e intervenciones que contribuyan a su salud sexual y elecciones reproductivas.

## Destacados

**(1)** Se observó un alto puntaje de autonomía reproductiva incluso durante la pandemia.

**(2)** No tener pareja estable influyó positivamente en la toma de decisiones reproductivas.

**(3)** Las mujeres de la capital, de menor franja etaria y con menor educación, tienen menos coerción.

**(4)** Ser nuligesta y utilizar anticonceptivos presentan menos coerción y mejor comunicación.

**(5)** El uso de un instrumento *online* durante la pandemia amplió el alcance a las mujeres.

## Introducción

Los derechos reproductivos de las mujeres se lograron después de las movilizaciones sociales y feministas que comenzaron en la década de 1960 y transformaron la sexualidad y beneficiaron la conformación social de las mismas. Sin embargo, a pesar de sus victorias, los límites impuestos por la desigualdad de género persisten en la vida cotidiana de las mujeres y continúan afectando su autonomía reproductiva^(1)^.

Garantizar la autonomía reproductiva es una tarea compleja, que involucra múltiples factores, entre los que se encuentran el contexto de vida y las necesidades individuales de las mujeres, factores socioeconómicos y demográficos, como edad, vivienda, años de educación, religión, estado civil, color, entre otros condicionantes y determinantes de la salud^(2-3)^.

La diferencia entre géneros también puede influir en que elijan o no utilizar anticonceptivos, considerando que las mujeres tienen mayor conocimiento sobre los métodos anticonceptivos disponibles y que los hombres ejercen una fuerte influencia en la planificación reproductiva^(4)^. En la elección de la anticoncepción hay que considerar el contexto de vida, la participación de la pareja en ese proceso y la emancipación y autonomía reproductiva de la mujer^(2)^.

Se destaca la importancia y la libertad de elección de la anticoncepción moderna para las mujeres y sus parejas sexuales, si es aceptable y segura dentro de las particularidades clínicas detectadas en la consulta con el profesional de la salud. Aquellas que no deseen quedar embarazadas, deberán iniciar o continuar con el método anticonceptivo elegido, eso garantiza sus derechos y su salud sexual y reproductiva^(5)^. La autonomía reproductiva les da a las mujeres el pleno derecho a ser protagonistas de sus elecciones anticonceptivas, ya sea que deseen o no tener hijos y utilizar o no métodos anticonceptivos^(6)^.

La pandemia de SARS-CoV-2 (COVID-19), principalmente en el período de 2020 a 2021, le impuso muchas restricciones a la población, debido a la necesidad de prevenir y reducir la propagación de la infección, a través del aislamiento social. Las restricciones también provocaron una reducción de la disponibilidad de los profesionales de la salud y generó enfermedades y estrés laboral, que se reflejaron en la calidad y resolución de los servicios de salud^(7)^.

En lo que respecta a la planificación reproductiva, el distanciamiento físico, el trabajo desde casa y las restricciones impuestas a las actividades no esenciales dificultaron el acceso a los servicios de salud sexual y reproductiva. Esto puede haber afectado los derechos y la autonomía de las mujeres y hacerlas más propensas a embarazos no planificados^(8-9)^.

El acceso a métodos anticonceptivos tiene un efecto positivo en la vida, la salud y el bienestar de las mujeres y sus familias, además reduce los embarazos no planificados^(10)^. La autonomía reproductiva de las mujeres brasileñas es de nivel medio^(11)^ a alto^(12)^, sin embargo, ellas aún enfrentan dificultades relacionadas con factores socioeconómicos y el papel de la pareja en el proceso de decisión reproductiva. Por ende, el papel del género y el poder en las decisiones son brechas que requieren mayor discusión^(6)^, al igual que los factores socioeconómicos y culturales, que se destacan con el mismo grado de importancia e influencia, según un estudio realizado en un país en desarrollo. Este se centró en adolescentes y mujeres, en las cuales la presencia de estigma se destacó como elemento que debe ser investigado^(13)^, y ese es un vacío en los estudios con mujeres brasileñas.

La violación de los derechos reproductivos y las imposiciones sociales comprometen la autonomía y pueden dificultar que las mujeres brasileñas alcancen sus objetivos reproductivos^(11)^. Por ende, este tema se eligió porque brinda la oportunidad de reflexionar sobre la autonomía reproductiva de las mujeres durante la pandemia de COVID-19, considerando también los cambios que se generaron en ese período y la influencia de los determinantes sociales de la salud.

El presente estudio tiene como objetivo contribuir a lograr una mejor comprensión de la autonomía reproductiva y si se conservó durante la pandemia de COVID-19. Por lo tanto, su objetivo fue analizar la autonomía reproductiva de las mujeres durante la pandemia de COVID-19, en función de factores sociodemográficos, clínicos y reproductivos.

## Método

### Diseño del estudio

Este es un estudio cuantitativo con un diseño transversal.

### Escenario

El estudio abarcó el estado de Alagoas, Brasil. Está ubicado en la región nordeste y está compuesto por 102 municipios, Alagoas tiene una población estimada de 3.365.351 habitantes y se divide en dos macrorregiones sanitarias. La primera macrorregión comprende de la primera a la sexta región sanitaria y abarca 57 municipios (2.276.293 habitantes), entre los que se encuentra la capital Maceió. A su vez, la segunda macrorregión sanitaria comprende de la séptima a la décima región e incluye en total 45 municipios (1.089.058 habitantes)^(14)^.

### Período

La recolección de datos se realizó del 24 de junio al 30 de noviembre de 2021.

### Población

La población de estudio estuvo compuesta por mujeres residentes en el estado de Alagoas, con edades entre 18 y 49 años.

### Criterios de selección

Los criterios de inclusión fueron: mujeres entre 18 y 49 años residentes en el estado de Alagoas. A su vez, los criterios de exclusión fueron: mujeres cuyos instrumentos de recolección de información estuvieran incompletos, duplicados o que no tuvieran correo electrónico o *WhatsApp* ^®^ para enviarles el Formulario de Consentimiento Libre e Informado (FCLI).

### Definición de la muestra

Para obtener la muestra del estudio, se realizó una búsqueda en el departamento de informática del Sistema Único de Salud (DATASUS) sobre la población residente en el estado de Alagoas, a saber: mujeres, en edad fértil, en la franja etaria de 18 a 49 años, por macrorregión de salud y en el período de 2010, año del último censo realizado por el Instituto Brasileño de Geografía y Estadística (IBGE).

La investigación incluyó un total de 777.970 mujeres, 536.820 mujeres de la primera macrorregión sanitaria y 241.150 mujeres de la segunda macrorregión sanitaria. A partir de esos datos se estimó la muestra del estudio mediante cálculo muestral, utilizando un margen de error del 5% y un nivel de confianza del 95%, y se obtuvo como resultado un total de 384 participantes.

Luego de aplicar los criterios de inclusión y exclusión, fueron incluidas en total 314 mujeres, cuyos instrumentos estaban completos, se tomó como referencia un estudio realizado en el nordeste de Brasil^(12)^. Para minimizar el sesgo de la muestra, la estrategia de difusión se dirigió a los municipios donde se obtuvieron menos respuestas a los instrumentos para recolectar información *online*. Cabe señalar que, inicialmente, participaron en el estudio 404 mujeres. Al excluir los instrumentos incompletos (n=62), los respondidos por la misma participante (n=12), aquellos cuya respuesta era incompatible con la pregunta sobre la edad (n=14) y los que no contaban con correo electrónico o *WhatsApp* ^®^ para enviar el FCLI (n=2), se obtuvo una muestra final de 314 mujeres.

### Variables del estudio

Se recolectaron datos sociodemográficos, clínicos y reproductivos mediante 46 variables, que se utilizaron para caracterizar a las participantes. Datos sociodemográficos: ciudad, edad, color (autodeclarado), pareja sexual actual, religión, ingreso mensual, educación de la participante y del jefe de familia, si tiene seguro médico y si recibe ayuda del gobierno. Para facilitar el análisis de los datos, las alternativas para la variable pareja actual se resumieron en dos grupos: sin pareja (soltera, viuda o divorciada) o con pareja sexual (casada o en unión estable).

A las variables sociodemográficas se agregaron 15, según los Criterios Brasileños de la Asociación Brasileña de Empresas de Investigación^(15)^ que evalúa la clasificación económica de Brasil, y están compuestas por variables cuyas opciones de respuesta son: cero, uno, dos, tres y cuatro o más. Las variables son: número de baños, empleadas domésticas que cobran un salario mensual y trabajan permanentemente cinco o más días por semana y autos de uso personal o familiar en la residencia donde vive la participante; número de microcomputadoras, lavavajillas, lavadoras y secadoras, refrigeradores, freezers, reproductores de Discos Versátiles Digitales (DVD), microondas y motocicletas en la residencia donde vive la participante; si hay agua corriente y pavimento en la calle donde vive la participante.

Cada respuesta (cero, uno, dos, tres y cuatro o más), dependiendo de la variable, corresponde a un número de puntos que van del 0 al 14. El puntaje total se obtiene sumando los puntos de cada variable, lo que da como resultado un punto de corte del Criterio Brasil según el ingreso medio: clase A (R$ 21.826,74) = 45 a 100 puntos; clase B1 (R$ 10.361,48) = 38 a 44 puntos; clase B2 (R$ 5.755,23) = 29 a 37 puntos; clase C1 (R$ 3.276,76) = 23 a 28 puntos; clase C2 (R$ 1.965,87) = 17 a 22 puntos; y clase DE (R$ 900,60) = 0 a 16 puntos. El ingreso promedio más alto corresponde a la clase “A” y el más bajo a la clase “DE”, y va bajando progresivamente^(15)^. Las demás variables sociodemográficas fueron creadas por las autoras, al igual que las variables clínicas y reproductivas.

Datos sobre condiciones clínicas: presencia de dislipidemia, hipertensión arterial, diabetes, obesidad, sedentarismo, tabaquismo u otros. Datos sobre cuestiones reproductivas: número de embarazos, incluidos los abortos; tener hijos, cantidad y intervalo de tiempo entre ellos y si todavía planea tener hijos; planificación del embarazo; qué métodos anticonceptivos conoce, cuáles ha utilizado, cuáles utilizaba antes de la pandemia de COVID-19 y en el momento de la investigación, cuál le gustaría utilizar; cuánto tiempo lleva usando el método anticonceptivo actual; si acudió a algún servicio o asistencia profesional de planificación reproductiva durante la pandemia (asesoramiento reproductivo); de quién recibió orientación sobre métodos anticonceptivos; forma de acceso, si paga por el método anticonceptivo y si el valor afecta sus ingresos; si cree que debido a la pandemia la elección de anticonceptivos se vio afectada; si antes de la pandemia tenía dificultades de acceso y si tiene o tuvo dificultades durante la misma; si interrumpió el método anticonceptivo durante la pandemia y por qué lo hizo.

La autonomía reproductiva fue evaluada mediante la *Reproductive Autonomy Scale* traducida, validada y adaptada para mujeres brasileñas^(16)^, que consta de 14 variables y está organizada en tres subescalas que serán presentadas en la sección sobre instrumentos utilizados para la recolección de información.

### Instrumentos utilizados para recolectar información

Los datos fueron obtenidos a través del cuestionario de evaluación de datos sociodemográficos, clínicos y reproductivos y de la Escala de Autonomía Reproductiva – versión brasileña^(16)^. El cuestionario consta de 46 preguntas. La Escala de Autonomía Reproductiva – versión brasileña^(16)^ está compuesta por 14 ítems, organizados en tres subescalas: toma de decisiones, ausencia de coerción y comunicación.

La subescala toma de decisiones consta de cuatro preguntas sobre quién tiene la última palabra en situaciones reproductivas: (1) quién decide si utilizas un método para evitar el embarazo; (2) quién decide qué método usarías para prevenir el embarazo; (3) quién decide cuándo tienes un bebé; (4) si quedas embarazada, de forma no planificada, ¿quién decidirá qué hacer?, ya sea criar al niño, buscar padres adoptivos o abortar. Para esta subescala se obtiene una puntuación según las respuestas: mi pareja sexual = 1 punto; yo y mi pareja sexual por igual = 2 puntos; yo = 3 puntos. Se les indica a las participantes que consideren y respondan al ítem “pareja sexual” a la pareja principal, la pareja más reciente o alguien de la familia que tendría alguna influencia en la decisión, como los padres o los suegros^(16-17)^.

La subescala de ausencia de coerción está compuesta por cinco preguntas sobre situaciones de coerción que sufren las mujeres: (1) tu pareja te impidió utilizar un método para evitar el embarazo cuando tú querías utilizar uno; (2) tu pareja te obstaculizó o dificultó el uso de un método para prevenir el embarazo cuando tú querías utilizar uno; (3) tu pareja te obligó a usar un método para evitar el embarazo cuando tú no querías usarlo; (4) tu pareja te impediría utilizar un método para prevenir el embarazo si tú quisieras utilizar uno; (5) tu pareja te presionó para quedar embarazada^(16)^.

La subescala de comunicación está compuesta por cinco afirmaciones sobre la capacidad para comunicarse sobre la relación sexual y las decisiones reproductivas: (1) tu pareja te apoyaría si quisieras utilizar un método para evitar el embarazo; (2) es fácil hablar de sexo con tu pareja; (3) si no quisieras tener relaciones sexuales, podrías decírselo a su pareja; (4) si tuvieras dudas sobre estar embarazada o no estar embarazada, podrías hablar con tu pareja al respecto; (5) si realmente no quisieras quedar embarazada podrías convencer a tu pareja de no tener un hijo^(16)^.

Para la segunda y tercera subescala, las opciones de respuesta son del tipo Likert (muy en desacuerdo, en desacuerdo, de acuerdo o muy de acuerdo), con una puntuación de 1 a 4 puntos. Para la segunda subescala, es necesario invertir la puntuación de los ítems para calcular el puntaje de ausencia de coerción, ya que todos los ítems son teóricamente contrarios a la autonomía reproductiva^(16)^.

Después de sumar los puntajes de las tres subescalas de autonomía reproductiva, se calcula una puntuación media para la escala. Los puntajes medios más altos indican mayores niveles de autonomía reproductiva^(16-17)^. El puntaje de autonomía reproductiva total de la escala oscila entre 1,00 y 4,00, una media de 1,00 a 2,00 significa autonomía baja, de 2,01 a 3,00 autonomía media y de 3,01 a 4,00 autonomía alta^(11-12)^.

### Recolección de datos

La recolección de datos se realizó de forma remota, debido a la pandemia de COVID-19 y para poder llegar a las mujeres de todos los municipios, a través de la herramienta *Google Forms*, mediante invitación abierta en las redes sociales *Instagram* ^®^ y *Facebook* ^®^, correo electrónico y *WhatsApp* ^®^. Se crearon *flyers* con información destacada para despertar el interés de las invitadas de continuar leyendo el texto adjunto, que contiene la presentación de las investigadoras, el objetivo del estudio, la población objetivo y el *link* para acceder a los instrumentos de recolección de información.

*Instagram* ^®^ permite compartir fotos, vídeos y mensajes. Además de utilizar las cuentas personales de las investigadoras, se creó un perfil específico para dar a conocer la investigación e invitar a las participantes. Los medios utilizados fueron historia, publicación en el *feed* y mensaje directo. Además, para llegar al mayor número posible de mujeres, se estableció contacto directo con perfiles activos, con un número importante de seguidores y público objetivo y contenidos dirigidos a residentes de los distintos municipios de Alagoas. Además, se contactó por *WhatsApp* ^®^ a los administradores de los perfiles que tenían los números disponibles en la biografía de *Instagram* ^®^.

La divulgación en *Facebook* ^®^ se realizó en las cuentas personales de las investigadoras, en las que se compartieron *flyers* y textos adjuntos. Se utilizó el correo electrónico institucional, ya que permitía un fácil acceso a los distintos correos electrónicos de estudiantes y profesores, conocidos o desconocidos, de la universidad donde estudiaban y trabajaban las investigadoras. *WhatsApp* ^®^ es una aplicación que se utiliza con el mismo fin que *Instagram* ^®^, con mensajes a través de chats grupales y mensajes privados.

Cabe señalar que todas las redes sociales fueron utilizadas para la difusión diaria durante el período de recolección de datos, además del mensaje adicional que solicitaba compartir el contenido recibido. No hubo distinción de género a la hora de enviar los mensajes de divulgación, junto al texto promocional había un mensaje sobre la importancia de compartir la información con una mujer que cumpliera con los criterios, si el destinatario no era parte del público objetivo. En el último mes de recolección de datos, las invitaciones se enviaron a los municipios donde se obtuvieron menos respuestas a los instrumentos de recolección de información.

La cumplimentación *online* del cuestionario de evaluación de datos sociodemográficos, clínicos y reproductivos y de la Escala de Autonomía Reproductiva - versión brasileña^(16)^ se realizó en días y horarios determinados por la disponibilidad de las participantes.

A través de la invitación abierta, al acceder al *link* de *Google Forms*, las participantes leyeron y conocieron la naturaleza de toda la investigación. Aceptaron participar en el estudio haciendo clic en el botón “ACEPTO” del FCLI, enviado individualmente, con copia de las respuestas, al correo electrónico o al número de *WhatsApp* ^®^ proporcionado al inicio del instrumento de recolección, para el archivo y/o o impresión de los documentos.

Poco después, las participantes respondieron a los instrumentos de la investigación para recolectar información. El tiempo de respuesta fue, en promedio, de quince a veinte minutos. En caso de dudas, fue posible resolverlas contactando a las investigadoras por correo electrónico. Para reclutar participantes se utilizó la técnica no probabilística, mediante la cual fueron incluidas las que aceptaron participar en el estudio y cumplieron los criterios de inclusión.

### Procesamiento y análisis de datos

Los datos fueron importados al programa *Microsoft Excel* para *Windows*, con el fin de organizar los datos y expresar los resultados de medias, desviaciones estándar y valores mínimos y máximos, con aplicación de estadística descriptiva. Para comparar las medias de los dominios de la Escala de Autonomía Reproductiva – versión brasileña – entre los grupos, se utilizó la prueba de Mann-Whitney para datos con distribución no paramétrica y la prueba T de Student para datos con distribución paramétrica. La normalidad de los datos se analizó mediante la prueba de Shapiro-Wilk, mientras que la homocedasticidad se analizó mediante la prueba de Levene. Se consideró un valor de p < 0,05 como nivel de significación. Los análisis se realizaron utilizando la versión del *Jeffreys’s Amazing Statistics Program* (JASP) versión 0.16.1.

### Aspectos éticos

El estudio respetó todos los aspectos éticos de acuerdo con la Resolución N° 466 de 2012 y la Resolución n° 510 de 2016 del Consejo Nacional de Ética en Investigación de Brasil (CONEP). La investigación fue aprobada bajo dictamen n° 4.794.176 el 21 de junio de 2021. Para aclarar dudas sobre el tema, después de completar la información se puso a disposición un *link* que redirige a la participante a una guía sobre Derechos Sexuales y Reproductivos de las Mujeres Durante la pandemia de COVID-19 elaborado por la Defensoría Pública del Estado de São Paulo y el Centro Especializado en Promoción y Defensa de los Derechos de la Mujer (NUDEM), que tiene orientaciones sobre anticoncepción, aborto legal, embarazo, parto y posparto^(18)^.

## Resultados

Se evaluaron las características sociodemográficas, clínicas y reproductivas de las participantes del estudio (n=314). El análisis de las características sociodemográficas mostró que la edad promedio de las mujeres era de 27,2 años. Además, el 87,26% (n=186) vivía en la capital de Alagoas; el 87,26% (n=274) se declaró no negra, es decir, el 37,58% (n=118) se declaró blanca, el 46,18% (n=145) morena, el 3,18% (n=10) amarilla y el 0,32% (n=1) indígena; el 12,74% (n=40) se declaró negra; y el 85,03% (n=267) de las mujeres tenía religión.

La mayor parte de la muestra del estudio estuvo compuesta por mujeres sin pareja sexual, es decir, solteras, viudas y divorciadas, 64,97% (n=204). El 98,41% (n=309) tenía más de ocho años de educación; y el 54,78% (n=172) de la muestra tenía ingresos mensuales superiores a un salario mínimo, el 25,16% (n=79) hasta dos salarios, el 18,79% (n=59) entre tres y cinco salarios y el 10,83% (n=59) =34) ganaba más de cinco salarios, mientras que el 45,22% (n=142) de las mujeres tenían un ingreso de hasta un salario mínimo. Según los Criterios de Clasificación Económica Brasileña^(12)^, el 32,80% (n=103) de las mujeres pertenecían a la clase “B2” (promedio R$ 5.755,23), seguida de la clase “C1” (promedio R$ 3.276,76) con el 19,11% (n =60). La minoría pertenece a la clase “A” (promedio R$ 21.826,74) con el 6,69% (n=21) y a la clase “DE” (promedio R$ 900,60) con el 7,32% (n=23).

Entre las condiciones clínicas que informó el 21,19% (n=68) de las mujeres, el 0,94% (n=03) presentó dislipidemia; el 3,43% (n=11), hipertensión arterial; el 0,31% (n=01), diabetes; el 4,98% (n=16), obesidad; el 7,17% (n=23), sedentarismo; el 0,94% (n=03), tabaquismo y el 3,43% (n=11) otras afecciones. Por ende, el 78,82% (n=253) de las mujeres negaron tener algún problema clínico.

En cuanto a las características reproductivas, el 64,65% (n=203) de las participantes nunca había estado embarazada (incluye abortos); el 20,70% (n=65) de las que ya habían quedado embarazadas informó que tuvo embarazo no planificado; y el 81,53% (n=256) utilizó métodos anticonceptivos durante la pandemia de COVID-19.

El método anticonceptivo más utilizado por las mujeres durante la pandemia fue el hormonal, que fue usado por el 60,51% (n=190), seguido del 41,72% (n=131) de los métodos de barrera y del 28,98% (n= 91) de los conductuales. El 45,22% (n=142) de las participantes llevaba más de dos años utilizando su método anticonceptivo. La mayoría de las participantes del estudio no interrumpió el uso de anticonceptivos durante la pandemia de COVID-19, 74,52% (n=234) de la muestra.

Además, el 61,47% (n=193) de las mujeres tuvo acceso a anticonceptivos mediante la compra con su propio dinero y el 87,26% (n=274) no tuvo dificultades para acceder a ellos durante la pandemia. Sin embargo, el 67,20% (n=211) de las mujeres no acudió a un servicio o atención profesional de planificación reproductiva durante la pandemia de COVID-19.

Las medias, desviaciones estándar y puntajes mínimos y máximos para los dominios de autonomía reproductiva se presentan en la Tabla 1. Se observó mayor autonomía en los constructos “Ausencia de coerción” y “Comunicación” y menor autonomía en “Toma de decisiones”.

**Tabla 1:** - Puntajes de autonomía reproductiva de las participantes del estudio, según cada dominio de la Escala de Autonomía Reproductiva (N* = 314). Maceió, AL, Brasil, 2021

**Factor (subescala)**	**Media**	**Desviación estándar**	**Mínimo - Máximo**
Toma de decisiones	2,65	0,51	1,00 - 3,00
Ausencia de coerción	3,63	0,66	1,00 - 4,00
Comunicación	3,41	0,75	1,00 - 4,00
Total	3,27	0,77	1,00 - 4,00

^*^N = Número total de participantes

Se analizaron los puntajes medios de autonomía reproductiva y las características sociodemográficas y reproductivas de las participantes del estudio, como se muestra en la Tabla 2. Se encontró diferencia estadísticamente significativa entre los puntajes medios de “Ausencia de coerción” y “Comunicación” y la franja etaria. Según los análisis, las mujeres entre 18 y 35 años tuvieron mayor autonomía en ambos puntajes que las mujeres mayores de 35 años.

**Tabla 2:** - Comparación entre los puntajes medios de autonomía reproductiva y las características sociodemográficas y reproductivas de las participantes del estudio (N^*^ = 314). Maceió, AL, Brasil, 2021

**Variables**	**Toma de decisiones**	**Ausencia de coerción**	**Comunicación**
**Franja etaria (años)**			
18-35	2,66±0,35	3,65±0,55	3,45±0,50
> 35	2,59±0,41	3,50±0,50	3,19±0,61
Valor de *p*	0,37	*0,03*	*<0,00* ^†^
**Residencia**			
Capital	2,68±0,33	3,70±0,51	3,48±0,49
Interior	2,61±0,38	3,52±0,59	3,31±0,55
Valor de *p*	0,12	*<0,001* ^†^	*<0,01* ^†^
**Color autodeclarado**			
Negras	2,73±0,34	3,56±0,57	3,40±0,43
No negras	2,64±0,36	3,64±0,55	3,41±0,53
Valor de *p*	0,11	0,30	0,60
**Religión**			
Con religión	2,64±0,36	3,61±0,55	3,39±0,53
Sin religión	2,72±0,35	3,69±0,54	3,51±0,44
Valor de *p*	0,07	0,23	0,21
**Pareja sexual actual**			
Con pareja sexual	2,54±0,38	3,64±0,52	3,41±0,58
Sin pareja sexual	2,71±0,33	3,62±0,57	3,41±0,49
Valor de *p*	*<0,001*	0,96	0,56
**Ingreso**			
Hasta 1 salario mínimo^‡^	2,66±0,37	3,58±0,59	3,35±0,50
> 1 salario mínimo^‡^	2,64±0,34	3,67±0,51	3,46±0,54
Valor de *p*	0,36	0,31	0,08
**Educación**			
Hasta 8 años de estudio	2,75±0,43	2,92±0,81	2,72±0,77
> 8 años de estudio	2,65±0,36	3,64±0,54	3,42±0,51
Valor de *p*	0,38	*0,02*	*0,02*
**Embarazo**			
No	2,65±0,35	3,70±0,49	3,46±0,50
Sí	2,66±0,38	3,49±0,63	3,33±0,55
Valor de *p*	0,56	*<0,001* ^†^	*0,04* ^†^
**Anticoncepción**			
No	2,68±0,39	3,51±0,56	3,14±0,63
Sí	2,65±0,35	3,65±0,55	3,47±0,47
Valor de *p*	0,31	*0,02* ^†^	*<0,001*
**Asesoramiento reproductivo**			
No	2,66±0,36	3,62±0,56	3,37±0,55
Sí	2,62±0,35	3,63±0,53	3,50±0,45
Valor de *p*	0,22	0,93	0,09

Los valores se expresan como media ± desviación estándar; ^*^N = Número total de participantes; ^†^Prueba T de Student; ^‡^El salario mínimo en la recolección de datos era de R$ 1.100,00, Brasil, 2021

También se verificó que había diferencia estadísticamente significativa entre los puntajes medios de “Ausencia de coerción” y “Comunicación” y la residencia. Los análisis demostraron que las participantes que vivían en la capital de Alagoas tenían mayor autonomía reproductiva que las mujeres que vivían en el interior.

En cuanto a las características sociodemográficas, hubo diferencia estadísticamente significativa entre el puntaje medio de “Toma de Decisiones” y de pareja sexual actual. Los análisis mostraron que las mujeres que no tenían pareja (solteras, viudas y divorciadas) tenían mayor autonomía en el constructo “Toma de Decisiones” que el grupo de participantes con pareja sexual (casadas y en unión estable).

Se encontró que había diferencia estadísticamente significativa entre los puntajes medios “Ausencia de coerción” y “Comunicación” y la educación. Según los análisis, las participantes con más de ocho años de estudio mostraron mayor autonomía en los constructos “Ausencia de coerción” y “Comunicación” que las participantes con hasta ocho años de estudio.

En cuanto a las características reproductivas, hubo diferencia estadísticamente significativa entre los puntajes medios de “Ausencia de coerción” y “Comunicación” y de embarazo y anticoncepción. Según los análisis, las mujeres que nunca han estado embarazadas (incluye abortos) y que utilizaban métodos anticonceptivos durante la pandemia de COVID-19 tenían mayor autonomía reproductiva que las mujeres que ya estuvieron embarazadas (incluye abortos) y que no utilizaban anticonceptivos.

## Discusión

Durante el período de recolección de datos, la pandemia de COVID-19 afectaba a Brasil. En Alagoas, el número de casos confirmados y defunciones iba en aumento y la tasa de incidencia fue del 6,3% en junio y del 7,2% en noviembre de 2021^(19-20)^. Por otro lado, hubo flexibilización y vacunación para mayores de 18 años, a partir de agosto de 2021^(21)^, también se dio el retorno gradual del acceso a los establecimientos, dependiendo de la capacidad de ocupación de personas y del comprobante de vacunación^(22)^. Además de las implicancias de la pandemia de COVID-19, hay que considerar que las dificultades y los desafíos de las mujeres para ejercer su pleno derecho a la autonomía reproductiva dependen de los diferentes factores asociados a la práctica, como el contexto sociodemográfico y reproductivo^(6)^.

Las mujeres de este estudio tuvieron un puntaje medio alto de autonomía reproductiva total. La mayor autonomía se observó en los constructos “Ausencia de coacción” y “Comunicación” y la menor autonomía en “Toma de decisiones”. A diferencia de otros estudios brasileños que observaron mayor autonomía en “Toma de decisiones”^(11)^ y menor autonomía en “Comunicación”^(12)^. Por otro lado, fue similar al resultado de un estudio con mujeres norteamericanas, que también arrojó menor autonomía en el constructo “Toma de Decisiones”^(17)^.

Se sabe que los factores sociodemográficos contribuyen a la autonomía reproductiva de las mujeres^(6)^. Con respecto a la subescala “Toma de Decisiones” y la variable pareja sexual actual, se observó en este estudio que las mujeres que no tienen pareja sexual (solteras, viudas y divorciadas) tenían mayor autonomía reproductiva, hallazgo que se repite en el estudio con quilombolas brasileñas^(11)^ y difiere del estudio realizado con trabajadoras rurales brasileñas^(12)^. Además, una investigación realizada con mujeres nigerianas reveló que las decisiones de salud de la mayoría de las mujeres participantes las tomaba su pareja y que los factores asociados son índice de riqueza y educación^(23)^. Las intenciones reproductivas de las mujeres se ven influenciadas por la relación con su pareja sexual, debido a la injerencia que tiene la pareja en la anticoncepción y a la coerción reproductiva que sufren, lo que revela que están sometidas a las decisiones de la pareja, hecho reforzado por el patriarcado y aspectos culturales que las rodean^(17)^. La coerción y presión masculina refuerzan la jerarquía de género y están presentes desde la sexarca, momento en el que las mujeres se encuentran en la fase de construcción de la autonomía sexual^(24)^. En la mayor parte de los casos, el sometimiento de las decisiones femeninas se relaciona con el número de hijos de la pareja y con la dependencia financiera, a pesar de que la responsabilidad de la anticoncepción se centre en la mujer^(4)^.

La variable franja etaria demostró que las mujeres de 18 a 35 años tienen mayor autonomía reproductiva en los constructos “Ausencia de coerción” y “Comunicación” que las mujeres mayores de 35 años, ese hallazgo es similar al de un estudio que validó la escala de autonomía reproductiva y obtuvo esos datos en mujeres jóvenes^(17)^. Al tratarse de una muestra de mujeres con una edad media de 27,2 años, la capacidad de comunicación en las parejas más jóvenes puede ser más efectiva para la toma de decisiones reproductivas, lo que da como resultado una mayor autonomía reproductiva.

Además, el inicio de las relaciones sexuales cada vez más temprano, la confianza en la pareja como condición para el uso del preservativo y los riesgos de que la población sea más vulnerable a embarazos no deseados son factores que pueden tener consecuencias a largo plazo sobre la salud sexual y reproductiva de la población más joven^(25)^. Por otro lado, el principal motivo de las mujeres sexualmente activas para no utilizar métodos anticonceptivos es no querer hacerlo o no preocuparse por quedar embarazadas, mientras que las mujeres jóvenes tienen doble protección^(3)^.

En cuanto a la variable residencia, las participantes que vivían en la capital del estado de Alagoas demostraron mayor autonomía reproductiva en los constructos “Ausencia de coerción” y “Comunicación” en este estudio que las mujeres que vivían en el interior del estado. Mientras que un estudio realizado en Brasil con mujeres de zonas rurales destacó que las mujeres tenían dificultades para alcanzar sus objetivos reproductivos debido a los determinantes sociales^(12)^. Esto puede estar relacionado con el acceso a la información que tienen las mujeres con menores ingresos, educación y condiciones de vivienda. Las consecuencias de los factores sociodemográficos desfavorables junto con la dificultad para comunicarse con la pareja y la coerción que sufren pueden tener consecuencias para la salud y la vida de las mujeres, e impedir el desarrollo de la autonomía reproductiva. De forma similar, un estudio realizado con adolescentes de una favela de Río de Janeiro demostró que la falta de respeto y el silenciamiento de las opciones sexuales y reproductivas de las mujeres en situaciones de mayor vulnerabilidad puede conducir a embarazos no planificados y abortos inseguros^(24)^.

En lo que respecta a la variable educación, las mujeres con más de ocho años de estudio demostraron mayor autonomía reproductiva en los constructos “Ausencia de coerción” y “Comunicación” que las mujeres con menos de ocho años de estudio. El estudio realizado en Nigeria demostró que cuanto mayor es el nivel educativo, mayor es la capacidad de la mujer para tomar sus propias decisiones de salud sin la interferencia de la pareja sexual o decisión conjunta^(23)^.

El conocimiento sobre anticoncepción y reproducción producto del alto nivel educativo de la mayoría de las participantes puede haber contribuido a que tuvieran una comunicación efectiva con la pareja para lograr objetivos reproductivos, dado que es al hombre al que se le da el valor social de la toma de decisiones. Cabe destacar que el bajo nivel educativo de las mujeres y la consiguiente falta de conocimiento contribuyen a la jerarquía de género y a que se valoren las decisiones que toman los hombres^(23)^.

La autonomía en la subescala “Comunicación” se repite en otros estudios^(12-13,17)^, y revela que una mejor comunicación con la pareja sexual contribuye a la toma de decisiones reproductivas. La dificultad para imponerle su voluntad a la pareja de rechazar la actividad sexual o de solicitarle que use preservativo puede afectar los derechos sexuales y reproductivos de la mujer^(24)^. El nivel de confianza en la pareja es un factor esencial para el uso regular del preservativo y favorece las conductas sexuales de riesgo, dado que depende del tiempo y del tipo de relación^(26)^.

Aunque no se encontró diferencia estadísticamente significativa entre la característica sociodemográfica “color”, varios estudios que evaluaron su influencia en la autonomía reproductiva sugieren que las mujeres negras tienen mayores limitaciones derivadas de las consecuencias del racismo estructural, e impacta en las decisiones reproductivas^(12)^.

Un estudio con mujeres negras arrojó como resultado menor autonomía en las subescalas “Ausencia de coerción” y “Comunicación”^(17)^ y otro realizado con mujeres quilombolas, entre las cuales el 64,7% se declararon negras, destacó la influencia de los factores sociodemográficos y reproductivos^(11)^. Cabe señalar que sólo el 12,74% de las participantes en el presente estudio se declararon negras, lo que puede haber contribuido a la obtención de mejores resultados relacionados con la autonomía reproductiva.

En las variables embarazo y anticoncepción, las mujeres que nunca habían estado embarazadas (incluye abortos) y que utilizaban anticonceptivos durante la pandemia de COVID-19 mostraron mayor autonomía reproductiva en la subescala “Comunicación” que las mujeres que ya habían quedado embarazadas (incluye abortos) y que no habían utilizado anticonceptivos. La comunicación en la pareja es esencial en todos los ámbitos de la reproducción, incluso en el aborto. Un estudio sobre la perspectiva masculina sobre el tema destaca las diferencias que hay en las relaciones entre géneros de distintas clases sociales y cómo la autonomía reproductiva de las mujeres implica una relación compleja con quienes las rodean, lo que puede generar diferentes participaciones de la pareja en la decisión de llevar el embarazo a término o de interrumpirlo^(27)^.

Por lo tanto, el diálogo en la pareja es, también, fundamental para la anticoncepción. La capacidad de comunicarse con la pareja sexual trae beneficios evidentes y elementos importantes para la anticoncepción y la reducción de conductas de riesgo^(26)^. Es importante considerar la presión masculina sobre las elecciones de anticoncepción y la autonomía de las mujeres^(4)^. Las variables embarazo y anticoncepción mostraron mayor autonomía reproductiva en el constructo “Ausencia de coerción” en mujeres que nunca habían estado embarazadas (incluye abortos) y que utilizaban anticonceptivos durante la pandemia de COVID-19.

El hecho de que la mayoría de las participantes utilice métodos anticonceptivos hormonales hace más de dos años, comprados con su propio dinero, y que no tengan dificultad para acceder a ellos, sugiere que tienen autonomía y empoderamiento sobre las decisiones reproductivas. Sin embargo, hay que considerar que la mayoría de las participantes tiene una posición privilegiada, ya que el nivel educativo, los ingresos y las condiciones de vivienda son factores que contribuyen al acceso a los anticonceptivos. Pero a pesar de que tiene gran acceso a métodos hormonales, esas mujeres no acceden frecuentemente a métodos a largo plazo.

Además, es importante señalar cómo la cultura del patriarcado y los juicios de la sociedad pueden afectar las elecciones de las mujeres con respecto a la decisión de vivir o no la maternidad, que se ven reforzadas por la imposición de lo que significa ser mujer y la visión de que las mujeres son las únicas responsables del hogar y de sus hijos^(28)^. Por lo tanto, preservar el derecho a acceder a la anticoncepción contribuye a la reducción de embarazos no planificados y tiene un profundo impacto en la vida y la autonomía reproductiva de las mujeres^(10)^.

Aún, la discontinuidad de los servicios de salud y el acceso reducido a métodos anticonceptivos durante las restricciones de la pandemia de COVID-19 pueden causar repercusiones negativas en la salud de las mujeres^(10)^. Las limitaciones al acceso a información, servicios y anticonceptivos no contribuyen a lograr la autonomía de las mujeres^(8)^.

Cabe señalar que la decisión compartida entre la mujer y el profesional de la salud puede favorecer la elección calificada y la adherencia efectiva a los anticonceptivos, cuando se realiza a través de la acogida, respetando la autonomía y las preferencias de la mujer^(29)^. En este caso, si bien el presente estudio no identificó una diferencia estadísticamente significativa en la variable asesoramiento reproductivo, cabe destacar la importancia que tiene la planificación reproductiva, ya que el 67,20% de las participantes no acudió a un servicio o atención profesional para tal fin durante el Pandemia de COVID-19, lo que puede generar riesgos, como embarazos no planificados y baja adherencia a los métodos anticonceptivos más efectivos y de larga duración.

El estudio tuvo limitaciones en cuanto al tamaño de la muestra, dado que el 22% de los instrumentos recolectados fueron excluidos por mala cumplimentación (duplicidad, respuesta incompatible con la pregunta y falta de contacto para enviar el FCLI) e instrumentos incompletos, a pesar del alto nivel educativo de las participantes. El uso del cuestionario y de la Escala *online*, publicados en redes sociales que requieren acceso a internet, puede haber dificultado la participación de mujeres con bajos niveles educativos e ingresos. Además, el perfil de las participantes del estudio puede no representar la realidad de la población general.

El uso de un instrumento *online* favorece el acceso a mujeres que viven en zonas de difícil acceso y permite un mayor alcance en los municipios, considerando también las restricciones de movilidad experimentadas durante la pandemia de COVID-19.

Los resultados del estudio contribuyen a dilucidar la autonomía de las mujeres durante la pandemia y la relación con sus parejas sexuales, además, a compararla con factores sociodemográficos y reproductivos, lo que sirve de base para la creación y mejora de políticas públicas dirigidas a controlar dichos factores.

Los aportes de este estudio están dirigidos a los profesionales de la salud que tienen mayor contacto con la población, como es el caso de los enfermeros, especialmente aquellos que realizan planificación reproductiva. También es de interés para otros profesionales y áreas más allá de la salud, con el fin de cambiar la cultura de responsabilizar exclusivamente a las mujeres de la anticoncepción, así como también de planificar acciones estratégicas para incluir alianzas en este proceso.

## Conclusión

Este estudio evaluó la autonomía reproductiva de las mujeres durante la pandemia de COVID-19. Según los datos obtenidos, no tener pareja sexual (soltera, viuda y divorciada) influyó positivamente en la autonomía para tomar decisiones reproductivas durante la pandemia de COVID-19, sin embargo, este mismo grupo presentó menos autonomía reproductiva total en la subescala “Toma de decisiones”. Las mujeres que pertenecían a la menor franja etaria, residían en la capital del estado, tenían mayor educación, nunca habían estado embarazadas y utilizaban anticonceptivos durante la pandemia de COVID-19, presentaron mayor autonomía en ausencia de coerción y comunicación.

Sentirse cómoda hablando con su pareja sexual sobre temas sexuales y reproductivos puede influir en la autonomía de la mujer. Las mujeres de este estudio tuvieron un puntaje medio alto de autonomía reproductiva total. La Escala de Autonomía Reproductiva - versión brasileña contribuyó a comprender las intenciones reproductivas de las mujeres participantes y permitió identificar los grupos que necesitan mayor atención y proponer intervenciones en salud sexual y reproductiva.

# Translation: Autonomia reprodutiva de mulheres durante a pandemia da COVID-19: um estudo transversal^*^

**DOI:** 10.1590/1518-8345.6910.4438

**Authors:** Carolyne Souza de Moura Barbosa, Ana Cecília Silvestre da Silva, Géssyca Cavalcante de Melo, Alba Maria Bomfim de França

**Affiliations:** 1 Universidade Estadual de Ciências da Saúde de Alagoas, Maceió, AL, Brasil.; 2 Bolsista da Fundação de Amparo à Pesquisa do Estado de Alagoas (FAPEAL), Brasil.; 3 Universidade Estadual de Ciências da Saúde de Alagoas, Núcleo de Saúde Materno-Infantil e do Adolescente, Maceió, AL, Brasil.; 4 Maternidade Escola Santa Mônica, Unidade de Terapia Intensiva Neonatal, Maceió, AL, Brasil.; 5 Universidade Estadual de Ciências da Saúde de Alagoas, Núcleo de Ensino de Propedêutica e Terapêutica, Maceió, AL, Brasil.; 6 Secretaria Municipal de Saúde de Maceió, Diretoria de Vigilância em Saúde, Maceió, AL, Brasil.; 7 Centro Universitário de Maceió, Maceió, AL, Brasil.

**Keywords:** Autonomia Pessoal, Comportamento Reprodutivo, Saúde da Mulher, Anticoncepcionais, Planejamento Familiar, COVID-19

## Abstract

**Objetivo:**

analisar a autonomia reprodutiva das mulheres durante a pandemia da COVID-19, segundo fatores sociodemográficos, clínicos e reprodutivos.

**Método:**

estudo quantitativo com delineamento transversal, realizado com 314 mulheres de 18 a 49 anos. As informações foram obtidas por meio de questionário *online* contendo dados sociodemográficos, clínicos e reprodutivos e da Escala de Autonomia Reprodutiva. Foram utilizados os testes Mann-Whitney e t de Student para comparação entre as variáveis.

**Resultados:**

verificou-se diferença estatisticamente significativa entre os escores médios de “tomada de decisão” e estado conjugal (*p* = <0,001); e “ausência de coerção” e “comunicação” com faixa etária (*p* = 0,03 e <0,001), residência (*p* = <0,001 e <0,01), escolaridade (*p* = 0,02 e 0,02), gestação (*p* = <0,001 e 0,04) e contracepção (*p* = 0,02 e <0,001).

**Conclusão:**

não ter parceria sexual influenciou positivamente na autonomia para tomada de decisão reprodutiva durante a pandemia da COVID-19. Mulheres de menor faixa etária, residentes na capital, com maior escolaridade, que nunca gestaram e que utilizaram contraceptivos durante a pandemia apresentaram maior autonomia para ausência de coerção e comunicação. Foi possível identificar os grupos que necessitam de maior atenção e intervenções que contribuam para sua saúde sexual e escolhas reprodutivas.

## Destaques

**(1)** Observou-se um alto escore de autonomia reprodutiva mesmo durante a pandemia.

**(2)** Não possuir parceiro fixo influenciou positivamente a tomada de decisão reprodutiva.

**(3)** Mulheres da capital, de menor faixa etária e menor escolaridade, possuem menor coerção.

**(4)** Ser nuligesta e utilizar contracepção apresentam menor coerção e melhor comunicação.

**(5)** A utilização de instrumento online durante a pandemia ampliou o alcance às mulheres.

## Introdução

Os direitos reprodutivos das mulheres foram conquistados após mobilizações sociais e feministas que se iniciaram na década de 60, transformando a sexualidade e beneficiando a conformação social das mesmas. Entretanto, apesar de suas vitórias, os limites impostos pela desigualdade de gênero persistem no cotidiano feminino e continuam interferindo na autonomia reprodutiva dessas mulheres^(1)^.

Garantir a autonomia reprodutiva é uma tarefa complexa, que envolve múltiplos fatores, considerando o contexto de vida e as necessidades individuais das mulheres, fatores socioeconômicos e demográficos, como idade, moradia, anos de escolaridade, religião, estado conjugal, cor, entre outros condicionantes e determinantes de saúde^(2-3)^.

A diferença entre os gêneros também pode influenciar na escolha ou não por contracepção, considerando o maior conhecimento das mulheres sobre métodos contraceptivos disponíveis e a forte influência masculina no planejamento reprodutivo^(4)^. Deve ser considerado o contexto de vida ante a escolha por contracepção, a participação da parceria nesse processo e a emancipação e autonomia reprodutiva das mulheres^(2)^.

Evidencia-se a importância e a liberdade de escolha por contracepção moderna para as mulheres e suas parcerias sexuais, caso seja aceitável e seguro dentro de suas particularidades clínicas investigadas em consulta com um profissional da saúde. Para aquelas que não desejam engravidar, devem iniciar ou continuar o método contraceptivo de escolha, garantindo assim seu direito e sua saúde sexual e reprodutiva^(5)^. A autonomia reprodutiva fornece o pleno direito da mulher como protagonista de suas escolhas contraceptivas, seja para ter ou não ter filhos e usar ou não usar contracepção^(6)^.

A pandemia por SARS-CoV-2 (COVID-19), especialmente no período de 2020 a 2021, proporcionou muitas restrições à população devido à necessidade de prevenção e redução da propagação da infecção, por meio do isolamento social. As restrições também impactaram na redução dos profissionais de saúde, além de gerar adoecimento e estresse ocupacional, refletindo na qualidade e resolutividade dos serviços de saúde^(7)^.

No âmbito do planejamento reprodutivo, o distanciamento físico, o trabalho em casa e as restrições de atividades não essenciais dificultaram o acesso aos serviços de saúde sexual e reprodutiva. Isso pode ter afetado os direitos e autonomia das mulheres, tornando-as mais propensas a gravidezes não planejadas^(8-9)^.

O acesso à contracepção impacta positivamente na vida, na saúde e no bem-estar de mulheres e suas famílias, além de reduzir gravidez não planejada^(10)^. A autonomia reprodutiva das mulheres brasileiras apresenta-se como de médio^(11)^ a alto nível^(12)^, porém, dificuldades ainda são enfrentadas no que tange aos fatores socioeconômicos e à atuação do parceiro no processo de decisão reprodutiva. Assim, o papel de gênero e poder nas decisões são lacunas que necessitam de maiores discussões^(6)^, bem como os fatores socioeconômicos e culturais, os quais são apontados com o mesmo grau de importância e influência, segundo estudo realizado em um país em desenvolvimento que teve como foco adolescentes e mulheres, cuja presença do estigma foi salientada como elemento a ser aprofundado^(13)^, sendo esta uma lacuna em estudos com mulheres brasileiras.

A violação dos direitos reprodutivos e as imposições sociais comprometem a autonomia e podem dificultar o alcance dos objetivos reprodutivos de mulheres brasileiras^(11)^. Assim, a escolha por esse tema foi motivada pela chance de refletir sobre a autonomia reprodutiva das mulheres durante a pandemia da COVID-19, considerando também as mudanças geradas por esse período e a influência dos determinantes sociais da saúde.

O presente estudo visa contribuir para uma melhor compreensão acerca da autonomia reprodutiva e se esta foi preservada durante a pandemia da COVID-19. Dessa forma, seu objetivo foi analisar a autonomia reprodutiva das mulheres durante a pandemia da COVID-19, segundo fatores sociodemográficos, clínicos e reprodutivos.

## Método

### Delineamento do estudo

Trata-se de um estudo quantitativo com delineamento transversal.

### Cenário

O estudo foi realizado abrangendo o estado de Alagoas, Brasil. Localizado na região nordeste e composto por 102 municípios, Alagoas contém uma população estimada de 3.365.351 habitantes, sendo dividido em duas macrorregiões de saúde. A primeira macrorregião compreende da primeira até a sexta região sanitária, totalizando 57 municípios (2.276.293 habitantes), incluindo a capital Maceió. Por sua vez, a segunda macrorregião de saúde compreende da sétima até a décima região, totalizando 45 municípios (1.089.058 habitantes)^(14)^.

### Período

A coleta de dados foi realizada durante o período de 24 de junho a 30 de novembro do ano de 2021.

### População

A população do estudo foi composta por mulheres residentes do estado de Alagoas com idade entre 18 e 49 anos.

### Critérios de seleção

Os critérios de inclusão foram: mulheres com 18 a 49 anos e que residiam no estado de Alagoas. Por sua vez, os critérios de exclusão foram: mulheres cujos instrumentos de coleta de informações estavam incompletos, duplicados, bem como sem *e-mail* ou *WhatsApp* ^®^ para envio do Termo de Consentimento Livre e Esclarecido (TCLE).

### Definição da amostra

Para obtenção da amostra do estudo, foi realizada uma busca no departamento de informática do Sistema Único de Saúde (DATASUS) sobre a população residente do estado de Alagoas, sendo: do sexo feminino, em idade fértil, por faixa etária detalhada de 18 a 49 anos, segundo a macrorregião de saúde e no período de 2010, ano do último censo do Instituto Brasileiro de Geografia e Estatística (IBGE).

A pesquisa contemplou um total de 777.970 mulheres, das quais 536.820 pertenciam à primeira macrorregião de saúde e 241.150 à segunda macrorregião de saúde. A partir disso, a amostra do estudo foi estimada por cálculo amostral, utilizando 5% de margem de erro e nível de confiança de 95%, resultando em um total de 384 participantes.

Após adoção dos critérios de inclusão e exclusão, foram incluídas um total de 314 mulheres, cujos instrumentos estavam completos, tendo como referência um estudo realizado no nordeste do Brasil^(12)^. Para minimizar o viés de amostra, foi utilizada a estratégia de divulgação direcionada para os municípios onde houve menos respondentes dos instrumentos para coleta de informações *online*. Salienta-se que, inicialmente, 404 mulheres participaram do estudo. Ao excluir os instrumentos incompletos (n=62), os respondidos pela mesma participante (n=12), aqueles com resposta incompatível à pergunta sobre a idade (n=14) e os sem *e-mail* ou *WhatsApp* ^®^ para envio do TCLE (n=2), obteve-se, ao final, uma amostra de 314 mulheres.

### Variáveis do estudo

Os dados sociodemográficos, clínicos e reprodutivos foram coletados mediante 46 variáveis, sendo utilizados para caracterização das participantes. Dados sociodemográficos: cidade, idade, cor (autodeclarada), parceria sexual atual, religião, renda mensal, escolaridade da participante e do chefe da família, se possui plano de saúde e se recebe auxílio governamental. Para auxiliar na análise dos dados, as alternativas da variável parceria atual foram sintetizadas em dois grupos: sem parceria (solteiras, viúvas ou divorciadas) ou com parceria sexual (casadas ou em união estável).

Dentre as variáveis sociodemográficas, 15 foram adicionadas segundo o Critério Brasil da Associação Brasileira de Empresas de Pesquisa^(15)^ - que avalia a classificação econômica do Brasil - e são compostas por variáveis com opções de respostas: zero, um, dois, três e quatro ou mais. São elas: quantidade de banheiros, de empregadas domésticas mensalistas que trabalham permanentemente por cinco a mais dias por semana e de automóveis/carro para uso pessoal ou da família na residência em que a participante mora; número de microcomputadores, máquinas de lavar louça, lavar roupa e secar roupa, geladeiras, freezers, aparelhos de Disco Versátil Digital (DVD), micro-ondas e motocicletas na residência em que a participante mora; se existe água encanada e pavimentação na rua onde a participante mora.

Cada resposta (zero, um, dois, três e quatro ou mais), a depender da variável, corresponde a uma quantidade de pontos que variam de 0 a 14. Uma pontuação total é obtida através da soma dos pontos de cada variável, resultando em um corte do Critério Brasil conforme a renda média: classe A (R$ 21.826,74) = 45 a 100 pontos; classe B1 (R$ 10.361,48) = 38 a 44 pontos; classe B2 (R$ 5.755,23) = 29 a 37 pontos; classe C1 (R$ 3.276,76) = 23 a 28 pontos; classe C2 (R$ 1.965,87) = 17 a 22 pontos; e classe DE (R$ 900,60) = 0 a 16 pontos. A maior renda média corresponde à classe “A” e a menor à classe “DE”, regredindo progressivamente^(15)^. As outras variáveis sociodemográficas foram elaboradas pelas próprias autoras, assim como as variáveis clínicas e reprodutivas.

Dados sobre condições clínicas: presença de dislipidemia, hipertensão arterial, diabetes, obesidade, sedentarismo, tabagismo ou outras. Dados sobre questões reprodutivas: número de gestações, incluindo abortamentos; ter filhos, quantidade, intervalo de tempo e se ainda planeja ter filhos; planejamento das gestações; quais métodos contraceptivos conhece, quais já utilizou, quais utilizava antes da pandemia da COVID-19 e no momento da pesquisa, qual gostaria de utilizar, há quanto tempo utiliza o método contraceptivo atual; se frequentou algum serviço ou atendimento profissional de planejamento reprodutivo durante a pandemia (aconselhamento reprodutivo); de quem já recebeu orientação sobre métodos contraceptivos; forma de acesso, se paga pelo método contraceptivo e se o valor interfere na renda; se acredita que devido à pandemia a escolha contraceptiva foi afetada; se antes da pandemia tinha dificuldades de acesso e se durante tem ou teve dificuldades; se interrompeu o método contraceptivo durante a pandemia e qual o motivo de ter interrompido.

A autonomia reprodutiva foi avaliada através da *Reproductive Autonomy Scale* traduzida, validada e adaptada para mulheres brasileiras^(16)^, com 14 variáveis e organizadas em três subescalas que serão apresentadas na seção dos instrumentos utilizados para coleta de informações.

### Instrumentos utilizados para coleta de informações

Os dados foram obtidos por meio do questionário para avaliação dos dados sociodemográficos, clínicos e reprodutivos e da Escala de Autonomia Reprodutiva — versão brasileira^(16)^. O questionário é composto por 46 questões. A Escala de Autonomia Reprodutiva–versão brasileira^(16)^ é composta por 14 itens, organizados em três subescalas: tomada de decisão, ausência de coerção e comunicação.

A subescala tomada de decisão é constituída por quatro perguntas sobre quem tem a palavra final diante das situações reprodutivas: (1) quem decide sobre você usar um método para evitar a gravidez; (2) quem decide sobre qual método você usaria para evitar a gravidez; (3) quem decide sobre quando ter um bebê em sua vida; (4) se você engravidasse, de forma não planejada, quem decidiria o que fazer – seja criar a criança, seja procurar por pais adotivos, seja fazer um aborto. Para essa subescala, uma pontuação é obtida conforme as respostas: meu parceiro sexual = 1 ponto; ambos, eu e meu parceiro sexual igualmente, = 2 pontos; eu = 3 pontos. As participantes são instruídas a pensar e responder como “parceiro sexual” o parceiro principal, o mais recente ou alguém da família que teria alguma influência na decisão, como os pais ou sogro (a)^(16-17)^.

A subescala ausência de coerção é formada por cinco questões referentes às situações de coerção sofrida pelas mulheres: (1) seu parceiro te impediu de usar um método para evitar a gravidez quando você queria usar um; (2) seu parceiro atrapalhou ou dificultou o uso de um método para evitar a gravidez quando você queria usar um; (3) seu parceiro te fez usar algum método para evitar a gravidez quando você não queria utilizar um; (4) seu parceiro te impediria de usar um método para evitar a gravidez se você quisesse usar um; (5) seu parceiro te pressionou para engravidar^(16)^.

A subescala comunicação é composta por cinco afirmações referentes à capacidade de comunicação sobre o relacionamento sexual e as decisões reprodutivas: (1) seu parceiro te apoiaria se você quisesse usar um método para evitar a gravidez; (2) é fácil falar sobre sexo com seu parceiro; (3) se você não quisesse ter relação sexual você poderia dizer para seu parceiro; (4) se você estivesse na dúvida em estar grávida ou não estar grávida, você poderia conversar com seu parceiro sobre isso; (5) se você realmente não quisesse ficar grávida você poderia convencer seu parceiro a não ter filho^(16)^.

Para a segunda e a terceira subescalas, as opções de resposta são do tipo Likert (eu discordo muito, eu discordo, eu concordo ou eu concordo muito), com pontuação de 1 a 4 pontos. Para a segunda subescala, é necessário inverter a pontuação dos itens para calcular o escore de ausência de coerção, pois todos os itens são teoricamente contrários à autonomia reprodutiva^(16)^.

Após a soma dos três escores das subescalas de autonomia reprodutiva, uma pontuação média é computada para a escala. Escores médios maiores indicam níveis mais altos de autonomia reprodutiva^(16-17)^. O escore de autonomia reprodutiva total da escala varia de 1,00 a 4,00, sendo a média de 1,00 a 2,00 baixa autonomia, de 2,01 a 3,00 média autonomia e 3,01 a 4,00 alta autonomia^(11-12)^.

### Coleta de dados

A coleta de dados foi realizada remotamente, devido à pandemia da COVID-19 e à possibilidade de alcançar mulheres em todos os municípios, por meio da ferramenta *Google Forms*, mediante convite aberto nas redes sociais *Instagram* ^®^ e *Facebook* ^®^, *e-mail* e *WhatsApp* ^®^. Foram elaborados *cards* de divulgação com informações em destaque para despertar o interesse das convidadas em continuar a leitura do texto anexado, contendo a apresentação das pesquisadoras, objetivo do estudo, população-alvo e direcionamento para o link dos instrumentos para coleta de informações.

O *Instagram* ^®^ permite o compartilhamento de fotos, vídeos e mensagens. Além do uso das contas pessoais das pesquisadoras, foi criado um perfil específico para divulgação e convite das participantes. Os meios utilizados foram o *story*, publicação no *feed* e contato via *Direct*. Além disso, para chegar ao máximo de mulheres, foi realizado o contato via *Direct* com perfis ativos, com número relevante de seguidores, público-alvo e conteúdo direcionado para moradores dos diversos municípios alagoanos. Como, também, o contato pelo *WhatsApp* ^®^ com os administradores dos perfis que tinham disponíveis os números na biografia do *Instagram* ^®^.

A divulgação no *Facebook* ^®^ foi realizada nas contas pessoais das pesquisadoras, com compartilhamentos dos *cards* e textos anexos. Foi utilizado o *e-mail* institucional, pois dava acesso facilitado aos diversos *e-mails* de discentes e docentes, conhecidos ou não, da universidade de estudo e trabalho das pesquisadoras. O *WhatsApp* ^®^ é um aplicativo utilizado com a mesma finalidade do *Instagram* ^®^, com divulgações via *chat* em grupos e mensagens individuais.

Ressalta-se que todas as redes sociais foram utilizadas para divulgação diária durante o período de coleta de dados, além da mensagem adicional solicitando o compartilhamento do conteúdo recebido. Não houve distinção de gênero nos envios, junto ao texto de divulgação havia a mensagem sobre a importância de compartilhar com uma mulher que se encaixasse nos critérios, caso não fizesse parte do público-alvo. No último mês de coleta de dados, os convites foram direcionados para os municípios onde houve menos respondentes dos instrumentos para coleta de informações.

O preenchimento *online* do questionário para avaliação dos dados sociodemográficos, clínicos e reprodutivos e da Escala de Autonomia Reprodutiva — versão brasileira^(16)^ foi realizado em dias e horários segundo a disponibilidade das participantes. Por meio do convite aberto, ao acessarem o *link* do *Google Forms*, as participantes fizeram a leitura e tomaram ciência da natureza de toda a pesquisa. Concordaram em participar do estudo ao clicar no botão “EU CONCORDO” do TCLE, encaminhado individualmente, com uma cópia das respostas, para o *e-mail* ou para o número de *WhatsApp* ^®^ disponibilizado no início do instrumento de coleta, para arquivamento e/ou impressão dos documentos.

Logo após, as participantes respondiam aos instrumentos para coleta de informações da pesquisa. O tempo de resposta foi, em média, de quinze a vinte minutos. Em caso de dúvidas, era possível saná-las, contactando as pesquisadoras por *e-mail*. Foi utilizada a técnica não probabilística para o recrutamento das participantes, onde eram incluídas enquanto aceitavam participar do estudo e posteriormente passar pelos critérios de exclusão.

### Tratamento e análise dos dados

Os dados foram importados para a *Microsoft Excel* para *Windows*, para organização dos dados e expressar os resultados das médias, desvios-padrão e valores mínimo e máximo, com aplicação de estatística descritiva. Para a comparação das médias dos domínios da Escala de Autonomia Reprodutiva — versão brasileira entre os grupos, foram utilizados o teste de Mann-Whitney para dados com distribuição não paramétrica e o teste t de Student para dados com distribuição paramétrica. A normalidade dos dados foi analisada por meio do teste Shapiro-Wilk, enquanto a homocedasticidade, pelo teste de Levene. Foi considerado como nível de significância o valor de *p* < 0,05. As análises foram realizadas no *Jeffreys’s Amazing Statistics Program* (JASP) versão 0.16.1.

### Aspectos éticos

O estudo respeitou todos os aspectos éticos em consonância à Resolução n.º 466 de 2012 e Resolução n.^o^ 510 de 2016 do Conselho Nacional de Ética em Pesquisa (CONEP). A pesquisa foi aprovada sob o parecer 4.794.176 em 21 de junho de 2021. Para esclarecimentos de dúvidas sobre a temática, foi disponibilizado um link ao se concluir o preenchimento das informações, o qual redireciona a participante para um guia sobre Direitos Sexuais e Reprodutivos das Mulheres Durante a Pandemia da COVID-19. Este foi elaborado pela Defensoria Pública do Estado de São Paulo e pelo Núcleo Especializado de Promoção e Defesa dos Direitos das Mulheres (NUDEM), que contém orientações sobre contracepção, aborto legal, gestação, parto e pós-parto^(18)^.

## Resultados

Foram avaliadas as características sociodemográficas, clínicas e reprodutivas das participantes do estudo (n=314). A análise das características sociodemográficas mostrou que a idade média das mulheres foi de 27,2 anos. Além disso, 87,26% (n=186) moravam na capital de Alagoas; 87,26% (n=274) se autodeclararam não pretas, ou seja, 37,58% (n=118) se autodeclararam como brancas, 46,18% (n=145) pardas, 3,18% (n=10) amarelas ou 0,32% (n=1) indígenas; 12,74% (n=40) se autodeclararam pretas; e 85,03% (n=267) das mulheres possuíam religião.

A maioria da amostra do estudo foi constituída por mulheres sem parceria sexual, ou seja, solteiras, viúvas e divorciadas, totalizando 64,97% (n=204). Identificou-se que 98,41% (n=309) possuía mais de oito anos de estudo; e 54,78% (n=172) da amostra possuía renda mensal maior que um salário mínimo, sendo 25,16% (n=79) até dois salários, 18,79% (n=59) entre três e cinco salários e 10,83% (n=34) mais de cinco salários, restando 45,22% (n=142) das mulheres que possuíam renda de até um salário mínimo. Segundo o Critério de Classificação Econômica Brasil^(12)^, 32,80% (n=103) das mulheres pertenciam à classe “B2” (média R$ 5.755,23), seguida da classe “C1” (média R$ 3.276,76) com 19,11% (n=60). A minoria é pertencente à classe “A” (média R$ 21.826,74) com 6,69% (n=21) e à classe “DE” (média R$ 900,60) com 7,32% (n=23).

Dentre as condições clínicas que 21,19% (n=68) das mulheres referiram, 0,94% (n=03) possuía dislipidemia; 3,43% (n=11), hipertensão arterial; 0,31% (n=01), diabetes; 4,98% (n=16), obesidade; 7,17% (n=23), sedentarismo; 0,94% (n=03), tabagismo e 3,43% (n=11) outras condições. Portanto, 78,82% (n=253) das mulheres negaram possuir qualquer alteração clínica.

Quanto às características reprodutivas, 64,65% (n=203) das participantes nunca estiveram grávidas (incluindo abortamento); 20,70% (n=65) das que já engravidaram referiram gravidez não planejada; e 81,53% (n=256) usaram métodos contraceptivos durante a pandemia da COVID-19.

Os métodos contraceptivos mais utilizados pelas mulheres durante a pandemia foram os hormonais, com 60,51% (n=190) de uso, seguidos de 41,72% (n=131) dos métodos de barreira e 28,98% (n=91) dos comportamentais. Salienta-se que 45,22% (n=142) das participantes utilizavam seu método contraceptivo há mais de dois anos. A maioria das participantes do estudo não interrompeu o uso do contraceptivo durante a pandemia da COVID-19, totalizando 74,52% (n=234) da amostra.

Além disso, 61,47% (n=193) das mulheres tiveram acesso ao contraceptivo por meio da compra com dinheiro próprio e 87,26% (n=274) não tiveram dificuldade de acesso durante a pandemia. Porém, 67,20% (n=211) das mulheres não frequentaram um serviço ou atendimento profissional de planejamento reprodutivo durante a pandemia da COVID-19.

As médias, desvios-padrão e pontuações mínimas e máximas dos domínios de autonomia reprodutiva estão apresentados na Tabela 1. A maior autonomia foi observada nos constructos “Ausência de coerção” e “Comunicação” e menor autonomia em “Tomada de decisão”.

**Tabela 1:** - Escores de autonomia reprodutiva das participantes do estudo, de acordo com cada domínio da Escala de Autonomia Reprodutiva (N^*^ = 314). Maceió, AL, Brasil, 2021

**Fator (subescala)**	**Média**	**Desvio-padrão**	**Mínimo - máximo**
Tomada de decisão	2,65	0,51	1,00 - 3,00
Ausência de coerção	3,63	0,66	1,00 - 4,00
Comunicação	3,41	0,75	1,00 - 4,00
Total	3,27	0,77	1,00 - 4,00

*N = Número total de participantes

Foram analisados os escores médios de autonomia reprodutiva e as características sociodemográficas e reprodutivas das participantes do estudo, conforme apresentado na Tabela 2. Foi verificada diferença estatisticamente significativa entre os escores médios de “Ausência de coerção” e “Comunicação” com faixa etária. Segundo as análises, as mulheres que tinham idade de 18 a 35 anos apresentaram maior autonomia em ambos os escores, quando comparadas às mulheres com idade maior que 35 anos.

**Tabela 2:** - Comparação entre os escores médios de autonomia reprodutiva e as características sociodemográficas e reprodutivas das participantes do estudo (N^*^ = 314). Maceió, AL, Brasil, 2021

**Variáveis**	**Tomada de decisão**	**Ausência de coerção**	**Comunicação**
**Faixa etária (anos)**			
18-35	2,66±0,35	3,65±0,55	3,45±0,50
> 35	2,59±0,41	3,50±0,50	3,19±0,61
Valor de *p*	0,37	*0,03*	*<0,00* ^†^
**Residência**			
Capital	2,68±0,33	3,70±0,51	3,48±0,49
Interior	2,61±0,38	3,52±0,59	3,31±0,55
Valor de *p*	0,12	*<0,001* ^†^	*<0,01* ^†^
**Cor autodeclarada**			
Pretas	2,73±0,34	3,56±0,57	3,40±0,43
Não pretas	2,64±0,36	3,64±0,55	3,41±0,53
Valor de *p*	0,11	0,30	0,60
**Religião**			
Com religião	2,64±0,36	3,61±0,55	3,39±0,53
Sem religião	2,72±0,35	3,69±0,54	3,51±0,44
Valor de *p*	0,07	0,23	0,21
**Parceria sexual atual**			
Com parceria sexual	2,54±0,38	3,64±0,52	3,41±0,58
Sem parceria sexual	2,71±0,33	3,62±0,57	3,41±0,49
Valor de *p*	*<0,001*	0,96	0,56
**Renda**			
Até 1 salário mínimo^‡^	2,66±0,37	3,58±0,59	3,35±0,50
> 1 salário mínimo^‡^	2,64±0,34	3,67±0,51	3,46±0,54
Valor de *p*	0,36	0,31	0,08
**Escolaridade**			
Até 8 anos de estudo	2,75±0,43	2,92±0,81	2,72±0,77
> 8 anos de estudo	2,65±0,36	3,64±0,54	3,42±0,51
Valor de *p*	0,38	*0,02*	*0,02*
**Gestação**			
Não	2,65±0,35	3,70±0,49	3,46±0,50
Sim	2,66±0,38	3,49±0,63	3,33±0,55
Valor de *p*	0,56	*<0,001* ^†^	*0,04* ^†^
**Contracepção**			
Não	2,68±0,39	3,51±0,56	3,14±0,63
Sim	2,65±0,35	3,65±0,55	3,47±0,47
Valor de *p*	0,31	*0,02* ^†^	*<0,001*
**Aconselhamento reprodutivo**			
Não	2,66±0,36	3,62±0,56	3,37±0,55
Sim	2,62±0,35	3,63±0,53	3,50±0,45
Valor de *p*	0,22	0,93	0,09

Os valores são expressos como média ± desvio-padrão; ^*^N = Número total de participantes; ^†^Teste t de Student; ^‡^O salário mínimo na coleta de dados foi de R$ 1.100,00, Brasil, 2021

Diferença estatisticamente significativa entre os escores médios “Ausência de coerção” e “Comunicação” com residência também foi verificada. As análises mostraram que as participantes que moravam na capital de Alagoas apresentaram maior autonomia reprodutiva quando comparadas às mulheres que moram no interior.

Quanto às características sociodemográficas, verificou-se diferença estatisticamente significativa entre o escore médio de “Tomada de decisão” e a parceria sexual atual. As análises mostraram que mulheres que não tinham parceria (solteiras, viúvas e divorciadas) apresentaram maior autonomia no constructo “Tomada de decisão” quando comparadas ao grupo de participantes com parceria sexual (casadas e em união estável).

Foi verificada diferença estatisticamente significativa entre os escores médios “Ausência de coerção” e “Comunicação” com escolaridade. Segundo as análises, as participantes com mais de oito anos de estudo apresentaram maior autonomia nos constructos “Ausência de coerção” e “Comunicação”, comparadas com as participantes com até oito anos de estudo.

Quanto às características reprodutivas, verificou-se diferença estatisticamente significativa entre os escores médios de “Ausência de coerção” e “Comunicação” com gestação e contracepção. Segundo as análises, mulheres que nunca estiveram grávidas (incluindo abortamento) e que utilizaram métodos contraceptivos durante a pandemia da COVID-19 apresentaram maior autonomia reprodutiva, em relação às mulheres que já engravidaram (incluindo abortamento) e que não utilizaram contraceptivos.

## Discussão

Durante o período de coleta de dados, a pandemia da COVID-19 permanecia em curso no Brasil. Em Alagoas, o número de casos confirmados e óbitos estava em progressão e com taxa de incidência de 6,3% em junho e 7,2% em novembro de 2021^(19-20)^. Por outro lado, havia flexibilização e vacinação para maiores de 18 anos, a partir do mês de agosto de 2021^(21)^, além do retorno gradativo do acesso aos estabelecimentos, mediante capacidade de ocupação de pessoas e comprovação vacinal^(22)^. Para além das implicações da pandemia da COVID-19, deve-se considerar que as dificuldades e os desafios das mulheres em exercer o pleno direito da autonomia reprodutiva dependem dos vários fatores associados à prática, como o contexto sociodemográfico e reprodutivo^(6)^.

As mulheres deste estudo apresentaram um escore médio alto de autonomia reprodutiva total. A maior autonomia foi observada nos constructos “Ausência de coerção” e “Comunicação” e a menor autonomia em “Tomada de decisão”. Diferente de outros estudos brasileiros que apresentaram maior autonomia em “Tomada de decisão”^(11)^ e menor autonomia em “Comunicação”^(12)^. Por outro lado, foi semelhante ao estudo com norte-americanas que também indicou menor autonomia no constructo “Tomada de decisão”^(17)^.

É sabido que os fatores sociodemográficos contribuem para a autonomia reprodutiva das mulheres^(6)^. No que se refere à subescala “Tomada de decisão” e à variável parceria sexual atual, observou-se neste estudo que as mulheres que não têm parceria sexual (solteiras, viúvas e divorciadas) apresentaram maior autonomia reprodutiva, achado que se repete no estudo com quilombolas brasileiras^(11)^ e difere do estudo realizado com trabalhadoras rurais brasileiras^(12)^. Além dessas, uma pesquisa realizada com nigerianas mostrou que a maioria das mulheres participantes teve decisões de saúde tomadas pelo companheiro, tendo como fatores associados o índice de riqueza e a escolaridade^(23)^. Mulheres têm suas intenções reprodutivas influenciadas pelo relacionamento com sua parceria sexual, pela interferência da parceria na contracepção e coerção reprodutiva sofrida, revelando submissão às decisões da parceria, fortalecidas pelo patriarcado e aspectos culturais que as cercam^(17)^. A coerção e a pressão masculina reforçam a hierarquia de gênero e estão presentes desde a sexarca, na qual a mulher se encontra na fase de construção da autonomia sexual^(24)^. Em sua maioria, a submissão das decisões femininas está relacionada ao número de filhos do casal e à dependência financeira, apesar de a responsabilidade contraceptiva ser voltada à mulher^(4)^.

A variável faixa etária demonstrou que mulheres de 18 a 35 anos apresentam maior autonomia reprodutiva no constructo “Ausência de coerção” e “Comunicação”, comparado às mulheres com mais de 35 anos, similar ao estudo que validou a escala de autonomia reprodutiva, onde apontou esse dado em mulheres jovens^(17)^. Por se tratar de uma amostra com mulheres com idade média de 27,2 anos, a capacidade de comunicação entre casais mais jovens pode ser mais efetiva nas decisões reprodutivas, refletindo em maior autonomia reprodutiva.

Outrossim, a sexarca cada vez mais precoce, a confiança na parceria como condição para uso do preservativo e os riscos da população mais vulnerabilizada para gestações não planejadas são fatores que podem acarretar consequências a longo prazo na saúde sexual e reprodutiva da população mais jovem^(25)^. Por outro lado, mulheres sexualmente ativas que não utilizam métodos contraceptivos apresentam como motivo principal não querer ou não se importar em engravidar, ao mesmo tempo, em que mulheres jovens realizam mais dupla proteção^(3)^.

Quanto à variável residência, as participantes do estudo que moravam na capital do estado de Alagoas demonstraram maior autonomia reprodutiva nos constructos “Ausência de coerção” e “Comunicação”, quando comparadas às mulheres moradoras no interior do estado. Em consonância, um estudo realizado no Brasil com mulheres da zona rural evidenciou as dificuldades das mulheres em alcançar seu objetivo reprodutivo devido aos seus determinantes sociais^(12)^. Isso pode estar relacionado ao acesso à informação para mulheres com menores condições de renda, escolaridade e moradia. As consequências de fatores sociodemográficos desfavoráveis somados à dificuldade de comunicação com as parcerias e coerção sofrida podem gerar consequências para a saúde e vida das mulheres, impossibilitando o desenvolvimento da autonomia reprodutiva. Em acordo, estudo feito com adolescentes da favela do Rio de Janeiro mostrou que o desrespeito e o silenciamento das escolhas sexuais e reprodutivas das mulheres em situação de maior vulnerabilidade podem gerar gravidez não planejada e aborto inseguro^(24)^.

No tocante à variável escolaridade, as mulheres com mais de oito anos de estudo demonstraram maior autonomia reprodutiva nos constructos “Ausência de coerção” e “Comunicação”, comparadas com as mulheres com menos de oito anos de estudo. O estudo realizado na Nigéria mostrou que quanto maior o nível de escolaridade, maior a capacidade da mulher de tomar suas próprias decisões em saúde sem interferência da parceria sexual ou decisão conjunta^(23)^.

O conhecimento sobre contracepção e reprodução obtido a partir do alto nível de escolaridade da maioria das participantes pode ter contribuído para a comunicação efetiva com as parcerias para alcance dos objetivos reprodutivos, visto que ao homem é dado o valor social de tomada de decisão. Vale destacar que a baixa escolaridade e consequente falta de conhecimento da mulher contribuem com a hierarquia de gênero e valia da decisão masculina^(23)^.

A autonomia na subescala “Comunicação” se repete em outros estudos^(12-13,17)^, evidenciando que a comunicação facilitada com a parceria sexual contribui nas decisões reprodutivas. A dificuldade em impor a vontade à parceria para a recusa da prática sexual ou solicitação do uso do preservativo podem afetar os direitos sexuais e reprodutivos das mulheres^(24)^. O nível de confiança na parceria é fator essencial para o uso de preservativo regularmente e influencia os comportamentos sexuais de risco, sendo motivado pelo tempo e tipo de relacionamento^(26)^.

Embora não tenha sido verificada diferença estatisticamente significativa entre a característica sociodemográfica “cor”, vários estudos que avaliaram sua influência na autonomia reprodutiva sugerem que mulheres pretas possuem maiores limitações advindas das consequências do racismo estrutural, impactando nas decisões reprodutivas^(12)^.

Estudo com mulheres negras mostrou menor autonomia nas subescalas “Ausência de coerção” e “Comunicação”^(17)^ e outro realizado com mulheres quilombolas, dentre as quais 64,7% se autodeclaram pretas, destacou a influência dos fatores sociodemográficos e reprodutivos^(11)^. Vale salientar que apenas 12,74% das participantes do presente estudo se autodeclararam pretas, o que pode ter contribuído para resultados mais bem relacionados à autonomia reprodutiva.

Nas variáveis gestação e contracepção, mulheres que nunca estiveram grávidas (incluindo abortamento) e que utilizaram contraceptivos durante a pandemia da COVID-19 apresentaram maior autonomia reprodutiva na subescala “Comunicação”, em relação às mulheres que já engravidaram (incluindo abortamento) e que não utilizaram contraceptivos. A comunicação entre o casal é essencial em todas as esferas da reprodução, incluindo o abortamento. Um estudo sobre a perspectiva masculina frente ao assunto evidencia as diferenças nas relações entre gêneros de diferentes classes sociais e como a autonomia reprodutiva das mulheres envolve uma complexa relação com aqueles que as cercam, podendo gerar diferentes participações da parceria na decisão pela gestação a termo ou interrupção^(27)^.

Sendo assim, o diálogo entre casais é, da mesma forma, fundamental para a contracepção. A capacidade de comunicação com a parceria sexual é um benefício percebido e elemento importante na contracepção e redução dos comportamentos de risco^(26)^. É importante considerar a pressão masculina nas escolhas contraceptivas e autonomia das mulheres^(4)^. As variáveis gestação e contracepção evidenciaram maior autonomia reprodutiva no constructo “Ausência de coerção” em mulheres que nunca engravidaram (incluindo abortamento) e que faziam uso de contraceptivos durante a pandemia da COVID-19.

O fato de a maioria das participantes utilizar métodos contraceptivos hormonais há mais de dois anos, obtidos pela compra com dinheiro próprio e sem dificuldade de acesso, sugere autonomia e empoderamento sobre as decisões reprodutivas. Porém, deve-se considerar a posição privilegiada da maioria das participantes, visto que o grau de escolaridade, renda e condições de moradia são fatores contribuintes para o acesso aos contraceptivos. Todavia, apesar do grande acesso aos métodos hormonais, essas mulheres não acessam com frequência os métodos de longa duração.

Além disso, é importante pontuar sobre como a cultura do patriarcado e os julgamentos da sociedade podem afetar as escolhas das mulheres quanto à decisão sobre viver a maternidade ou não, fortalecidas pela imposição do que é ser mulher e pela visão de que as mulheres são as únicas responsáveis pelo lar e pelos seus filhos^(28)^. Ante o exposto, a preservação do direito de acesso à contracepção contribui na redução de gravidez não planejada e impacta profundamente na vida e na autonomia reprodutiva das mulheres^(10)^.

Outrossim, a descontinuidade dos serviços de saúde e a redução do acesso aos métodos contraceptivos durante as restrições da pandemia da COVID-19 podem causar repercussões negativas na saúde das mulheres^(10)^. Limitações ao acesso à informação, a serviços e a contraceptivos não contribuem para efetivar a autonomia da mulher^(8)^.

Salienta-se que a decisão compartilhada entre a mulher e um profissional de saúde pode favorecer a escolha qualificada e a adesão efetiva ao contraceptivo, ao ser realizada através do acolhimento, respeitando a autonomia e preferências da mulher^(29)^. Nesse contexto, apesar de o presente estudo não ter identificado diferença estatisticamente significativa na variável aconselhamento reprodutivo, convém destacar a importância do planejamento reprodutivo, uma vez que 67,20% das participantes não frequentaram um serviço ou atendimento profissional com tal finalidade durante a pandemia da COVID-19, podendo gerar riscos, como gravidez não planejada e baixa adesão aos métodos mais eficazes e de longa duração.

O estudo apresentou limitações quanto ao tamanho da amostra, pois 22% dos instrumentos coletados foram excluídos devido ao mau preenchimento (duplicidade, resposta incompatível com a pergunta e ausência de contato para envio do TCLE) e instrumentos incompletos, apesar do alto grau de escolaridade. A utilização do questionário e da Escala de forma *online*, divulgado em redes sociais que necessitam de internet para acesso, pode ter dificultado a participação de mulheres com baixa escolaridade e renda. Ademais, o perfil das participantes do estudo pode não representar a realidade da população em geral.

A utilização de um instrumento *online* promove o acesso às mulheres que residem em áreas de difícil acesso e possibilita maior alcance nos municípios, considerando também as restrições de deslocamento vivenciadas durante a pandemia da COVID-19.

Os resultados do estudo contribuem para elucidar sobre a autonomia da mulher durante a pandemia e a relação com suas parcerias sexuais, além da comparação com os fatores sociodemográficos e reprodutivos, servindo de suporte para construção e aprimoramento de políticas públicas direcionadas para o controle dos fatores mencionados.

As contribuições desse estudo são conduzidas aos profissionais da saúde que têm maior contato com a população, como a Enfermagem, sobretudo os que realizam planejamento reprodutivo. Interessa também a outros profissionais e áreas para além da saúde, com vistas às mudanças na cultura de responsabilização unicamente da mulher para contracepção, como também planejar ações estratégicas para inclusão das parcerias nesse processo.

## Conclusão

Este estudo avaliou a autonomia reprodutiva de mulheres durante a pandemia da COVID-19. Conforme os dados apresentados, não ter parceria sexual (solteiras, viúvas e divorciadas) influenciou positivamente na autonomia para tomada de decisão reprodutiva durante a pandemia da COVID-19, porém, esse mesmo grupo apresentou menor autonomia reprodutiva total na subescala “Tomada de decisão”. As mulheres de menor faixa etária, residentes na capital do estado, com maior escolaridade, que nunca gestaram e que utilizaram contraceptivos durante a pandemia da COVID-19, apresentaram maior autonomia para ausência de coerção e comunicação.

Sentir-se à vontade para conversar com sua parceria sexual sobre questões sexuais e reprodutivas pode influenciar a autonomia das mulheres. As mulheres deste estudo apresentaram um alto escore médio de autonomia reprodutiva total. A Escala de Autonomia Reprodutiva — versão brasileira contribuiu para compreensão das intenções reprodutivas das mulheres participantes, sendo possível identificar os grupos que necessitam de maior atenção e proposição de intervenções em saúde sexual e reprodutiva.
